# Interstitial flow potentiates TGF-β/Smad-signaling activity in lung cancer spheroids in a 3D-microfluidic chip†

**DOI:** 10.1039/d3lc00886j

**Published:** 2023-12-04

**Authors:** Zaid Rahman, Ankur Deep Bordoloi, Haifa Rouhana, Margherita Tavasso, Gerard van der Zon, Valeria Garbin, Peter ten Dijke, Pouyan E. Boukany

**Affiliations:** a Department of Chemical Engineering, Delft University of Technology Delft The Netherlands p.e.boukany@tudelft.nl; b Department of Cell and Chemical Biology and Oncode Institute, Leiden University Medical Center Leiden The Netherlands

## Abstract

Within the tumor microenvironment (TME), cancer cells use mechanotransduction pathways to convert biophysical forces to biochemical signals. However, the underlying mechanisms and functional significance of these pathways remain largely unclear. The upregulation of mechanosensitive pathways from biophysical forces such as interstitial flow (IF), leads to the activation of various cytokines, including transforming growth factor-β (TGF-β). TGF-β promotes in part *via* a Smad-dependent signaling pathway the epithelial–mesenchymal transition (EMT) in cancer cells. The latter process is linked to increased cancer cell motility and invasion. Current research models have limited ability to investigate the combined effects of biophysical forces (such as IF) and cytokines (TGF-β) in a 3D microenvironment. We used a 3D-matrix based microfluidic platform to demonstrate the potentiating effect of IF on exogenous TGF-β induced upregulation of the Smad-signaling activity and the expression of mesenchymal marker vimentin in A549 lung cancer spheroids. To monitor this, we used stably integrated fluorescent based reporters into the A549 cancer cell genome. Our results demonstrate that IF enhances exogenous TGF-β induced Smad-signaling activity in lung cancer spheroids embedded in a matrix microenvironment. In addition, we observed an increased cell motility for A549 spheroids when exposed to IF and TGF-β. Our 3D-microfluidic model integrated with real-time imaging provides a powerful tool for investigating cancer cell signaling and motility associated with invasion characteristics in a physiologically relevant TME.

## Abstract 2

3-D microfluidic platform to investigate effects of interstitial flow and TGF-β on A549 lung cancer spheroids.

## Graphical abstract

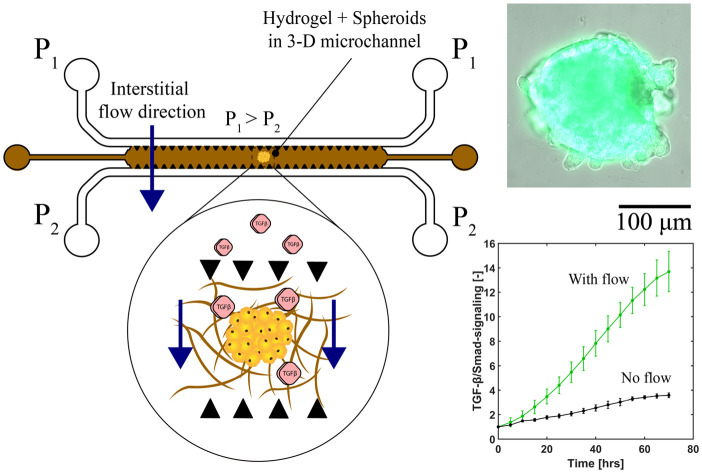

## 1 Introduction

The 3D tumor microenvironment (TME) plays a crucial role in the progression and metastasis of primary tumors to secondary tumor sites.^1,2^ It consists of key components such as the extracellular matrix (ECM), biophysical forces (interstitial flow and consequent fluid stresses), tumor cell–TME interactions in the presence of stromal cells, immune cells and cancer-associated fibroblasts (CAFs). The interplay of these components contributes to the metastatic cascade of events from early dissemination to extravasation.^3^ However, most tumor cell migration and invasion studies have been performed in 2D/3D *in vitro* models that poorly recapitulate the characteristics of solid tumors *in vivo*. To overcome these limitations, microfluidic platforms provide an effective tool to replicate a physiologically relevant TME for studying cancer cell behavior.^4^

Recent advances in microfluidic platforms based on a 3-D matrix have allowed for the incorporation of key components of the TME in cancer cell migration and invasion studies.^4,5^ To mimic the ECM, natural-hydrogel materials with tunable mechanical properties are used.^6^ These hydrogels have been further embedded with single cancer cells and/or cancer cell aggregate/spheroids to include cell–matrix interactions. Advancements in modeling and fabrication technologies have improved microfluidic devices to introduce interstitial flow (IF) for long-term perfusion and culture conditions. In the past, IF studies were mostly performed on single cells embedded in a matrix material.^7–9^ Recently, researchers have investigated the invasive and migratory cellular response of a breast tumor spheroid model under IF to show morphological and epigenetic changes.^10^ However, these studies were limited to highly migratory breast cancer cells and did not include effect of biochemical signals towards EMT signaling pathways.

The role of IF is important due to its direct influence on the remodeling of the ECM, where compressive, tensional^11–13^ and shear forces are sensed by cell-surface receptors that activate mechanotransduction pathways to trigger biochemical signals.^14,15^ This further leads to the activation and upregulation of many core EMT cytokines,^7^ including the TGF-β cytokine, known as a key EMT inducer.^16,17^ In solid tumors, the poorly drained IF is responsible for interstitial fluid pressure build up in the surrounding healthy tissue.^7^ Moreover, the lung tumor tissue is constantly subjected to a mechanical load due to its physiological activities that may aid in cancer cell invasion and migration.^18,19^ Therefore, it is of primary interest to study lung tumor models such as A549 lung adenocarcinoma, when subjected to biophysical force induced stresses. It has been proposed that the cancer cells exposed to biomechanical forces (such as IF, fluid-induced shear stress and compressive stress from matrix microenvironment) lead to endogenous TGF-β driven Smad-signaling activity towards EMT response.^15,20,21^ Moreover, studies have also investigated the role of fluid-induced shear stress to promote mechanotransduction pathways (such as YAP/TAZ) responsible for triggering EMT signaling for cancer cell invasion in non-small cell lung cancer, breast cancer and melanoma tumor.^22–24^

TGF-β is capable of promoting cancer cell invasion and progression in various tumor types such as lung, breast and pancreatic cancer.^25,26^ TGF-β receptors at the cell-surface upon binding TGF-β activate the intracellular Smad-signaling pathway.^25^ Activated Smads can act as transcription factors to mediate EMT associated with cancer. It is well known that the Smad-signaling pathway contributes to EMT, however Smad-independent pathways may also contribute to EMT *via* multiple complex intra-cellular signaling events that play an important role in cancer cell invasion.^27,28^ Many researchers have studied the role of TGF-β in static 2D/3D tumor models, highlighting its importance in activating EMT transcriptional factors including SNAIL, TWIST, ZEB1.^25^ Studies conducted on A549 lung adenocarcinoma cells showed EMT behavior upon exposure to TGF-β cytokine.^29–32^ Most studies focused on the upregulation of mesenchymal markers (such as vimentin) and an increased expression of transcription factors such as SNAIl and ZEB2 highlighting EMT response.^29,33,34^ The upregulation of the vimentin mesenchymal marker and downregulation of the E-cadherin (epithelial marker) in A549 lung cancer cells were found to be associated with an aggressive motile response.^26^ In recent years, researchers further studied A549 3D cancer models towards EMT behavior.^35,36^ However, these studies were performed in culture conditions devoid of matrix material and IF. Thus, there is an evident lack of research on the effect of IF and exogenous TGF-β on A549 lung tumor EMT response in a relevant matrix microenvironment.

**Fig. 1:**
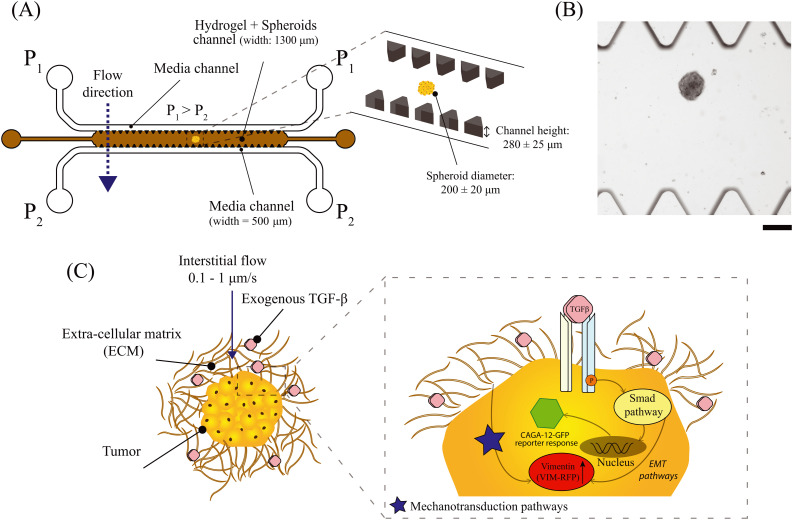
3D-matrix based microfluidic platform to study IF and TGF-β/Smad-signaling and vimentin expression in A549 lung tumor spheroids. (A) Schematic of the 3D-matrix based microfluidic platform. The inset figure shows the spheroid size and the dimensions of the channel height of the microfluidic chip (not to scale). (B) Bright-field image displaying A549 spheroid embedded in 3D-matrix based scaffold in the hydrogel channel of the microfluidic chip. Scale bar: 200 μm. (C) A549 lung tumor spheroid exposed to IF and exogenous TGF-β embedded in a matrix microenvironment. The inset figure illustrates that exogenous TGF-β molecules specifically bind with TGF-β surface receptors to activate the intracellular Smad-signaling pathway. This results in the upregulation of transcriptional reporter gene CAGA-12-GFP activity. TGF-β cytokine also leads to the upregulation in EMT pathways. Upregulation in EMT biomarker (vimentin) can be investigated by determining the VIM-RFP reporter intensity. Moreover, cancer cells sense IF induced fluid-shear and hydrodynamic stress to initiate mechanotransduction pathways triggering EMT. These biomechanical forces may enforce the TGF-β/Smad activity for increased transcriptional reporter activity (CAGA-12-GFP intensity) and EMT response (VIM-RFP reporter expression).

In this research, we employed a 3D-matrix based microfluidic model to investigate the impact of IF and exogenous TGF-β cytokine on epithelial-like A549 spheroids (Fig. 1A and B). Specifically, we investigated the Smad-dependent transcriptional pathway and vimentin biomarker expression in response to varying IF and exogenous TGF-β concentration towards cancer cell invasion (Fig. 1C). These studies were conducted with genetically modified A549 lung tumor cells with dual artificial reporter constructs for Smad-signaling pathway (CAGA-12-GFP reporter gene) and vimentin biomarker (VIM-RFP reporter gene) (Fig. 1C, inset figure). We demonstrate that IF potentiates Smad-dependent transcriptional reporter response when exposed to exogenous TGF-β. The combined effect of IF and TGF-β also showed an increase in the abundance of vimentin protein. Lastly, A549 lung tumor cells exhibited an increased cellular motion observed on the spheroid periphery characteristic for cancer cell invasion behavior. These findings suggest that external IF and cellular cues play critical roles in promoting the invasive characteristics of cancer cells within relevant matrix microenvironments, and highlight the importance of incorporating these factors in cancer research models.

## 2 Materials and methods

### 2.1 Cell culture

A549-VIM-RFP cells were acquired from the company, ATCC.^37^ These cells were engineered by CRISPR to produce a red fluorescent protein (RFP)–vimentin fusion protein. When cells acquire mesenchymal phenotype they express RFP linked to vimentin protein (VIM-RFP). To construct a dual reporter, A549-VIM-RFP cells were transduced with a lentiviral CAGA-12-GFP construct to produce a green fluorescent protein (GFP) response upon Smad-pathway activation. The dual reporter cell line was a gift from Yifan Zhu (Department of Cell and Chemical Biology, LUMC). The functionality of the CAGA-12-GFP reporter has been validated through several studies.^38,39^ A549-VIM-RFP cells have been previously reported to exhibit EMT with an upregulation in VIM-RFP fluorescence upon exogenous TGF-β stimulation.^40^ The dual-reporter A549 cells were maintained in Dulbecco's modified eagle medium high glucose (DMEM, Sigma) containing 4.5 g L^−1^ glucose, l-glutamine without sodium pyruvate, and supplemented with 10% fetal bovine serum (FBS, Sigma) and 1% antibiotic–antimycotic solution (Gibco). All cells were incubated at 37 °C with 5% CO_2_ and sub-cultured 2 times per week. Cells were frequently tested for absence of mycoplasma and checked for authenticity by STR profiling.

### 2.2 Spheroid fabrication

Spheroids were grown in a commercially available Corning™ Elplasia™ 96-well plate for high-throughput spheroid production. These well plates are round-bottom with ultra-low attachment (ULA) surface that prevents cell-surface attachment and promotes cell–cell adhesion. We used an initial seeding density of 40 × 10^3^ cells (500 cells per micro-well) for each well to produce 79 spheroids. Spheroid size is dependent on the initial seeding density, cell proliferation rate and culture duration. Spheroids were ready to use after 4 days of culture in the wells and were 200 ± 20 μm in diameter. We restricted the spheroid diameter to less than 220 μm to avoid a necrotic core and to avoid contact with the glass bottom of the microfluidic chip. Any cancer spheroids that made contact with the microfluidic glass substrate, were excluded from the analysis in this work.

### 2.3 Hydrogel synthesis and characterization

Gelatin methacryloyl (GelMA), 300 g bloom, 60% degree substitution, was purchased from Sigma Aldrich. Like gelatin, gelMA is still a thermo-reversible gel, however, the methacrylic anhydride groups give the ability to undergo covalent cross-linking under UV light (365 nm) in the presence of a UV photo-initiator. 5 wt% gelMA was used in experiments, with a mass ratio of 1 : 16 of photo-initiator (lithium phenyl-2,4,6-trimethylbenzoylphosphinate, LAP; Sigma Aldrich). LAP and gelMA were added together and dissolved in Dulbecco's phosphate buffered saline (DPBS; Gibco). The mixture was dissolved at 37 °C in a water bath for about 2 hours. The hydrogel was then crosslinked using a Colibri Axio Observer microscope laser 385 nm with a 5× objective lens for 45 seconds. The viscoelastic properties of crosslinked gelMA were investigated with a modular rotational rheometer (DSR 502, Anton Paar) equipped with a parallel plate of a diameter of 25 mm. Full experimental detail can be found in ESI† (section 1). The 5 wt% gelMA analyzed at a fixed strain of 1% with frequency sweeps (0.1 to 100 rad s^−1^) at room temperature showed a solid-like behavior, with a storage modulus *G*′ ≈ 250 Pa, higher than the loss modulus (*G*′′) by at least one order of magnitude (Fig. S1†). The lung tumor tissue stiffness is reported around 200 Pa *in vivo*.^41^ To replicate the mechanical properties of TME (under *in vivo* conditions), we employed a matrix material with similar mechanical properties.

### 2.4 Microfluidic chip fabrication and interstitial flow characterization

The microfluidic chip was fabricated on a 4 inch silicon wafer by the photo-lithography process in a cleanroom facility using μMLA Laser Writer (Heidelberg Instruments) (full procedure described in ESI,† section 2). The microfluidic chip design was inspired by IF studies performed with single cells and was upgraded to fabricate a channel height of 280 ± 25 μm (Fig. 1A).^42–44^ From the master mould, polydimethylsiloxane (PDMS) based microfluidic chips were fabricated by soft-lithography technique (refer to ESI,† section 2 for a detailed procedure).

The microfluidic chip consisted of three parallel channels separated by triangular pillars (all side lengths: 150 μm and height: 280 ± 25 μm). The middle channel was loaded with 5 wt% gelMA hydrogel, which is crosslinked under UV-light (385 nm) for 45 seconds. The top and bottom channels are the fluidic channels. The inlets of the top channel were maintained at a higher pressure (*P*_1_) relative to the bottom channel (*P*_2_) to generate an IF along the pressure gradient (Fig. 1A). By controlling the pressure of the reservoirs at (*P*_1_) and (*P*_2_), we were able to establish a pressure gradient to generate an IF through gelMA hydrogel across the microfluidic device. The inlet and outlet pressures were controlled by a pressure pump (Fluigent) and operated *via* InFlow software to pressurize the sample reservoirs. Fig. S2† shows the experimental setup for generating a continuous IF using a pressure pump device connected to the 3D-microfluidic chip. According to Darcy's law, flow velocity through a porous material is directly proportional to the pressure gradient governed by hydraulic permeability (*K*) of the material. In this case, we first calculated the hydraulic permeability of 5 wt% gelMA and then estimated the average IF velocity (*u*_m_); refer to ESI,† section 3 for detailed protocol. We tested two pressure drops (Δ*P* = *P*_1_ − *P*_2_) of 20 mbar and 30 mbar that corresponded to an interstitial flow velocity of *u*_m_ = 0.2 μm s^−1^ and 0.45 μm s^−1^ obtained *via* COMSOL Multiphysics using free and porous media flow interface (Fig. S3A and B†). The IF velocity calculated for our 3D-matrix based microfluidic system is physiologically relevant as previously reported. IF velocity in tumor tissues, performed *in vivo*, *in vitro* or *via* mathematical modelling are reported in the order of 0.01–1 μm s^−1^ in various cancer types.^7,45,46^ Moreover, several studies have highlighted the role of elevated interstitial fluid pressure (IFP) in a tumor tissue as a barrier to tumor treatment.^47^ IFP is reported in the order of 10 mbar to 60 mbar in various cancer types such as breast and melanoma skin cancer^47^ and other studies (modeling and *in vivo* experiments) have reported from 1–100 mbar.^45,48^

### 2.5 Microfluidic device setup for IF and exogenous TGF-β studies

To investigate the effect of IF and exogenous TGF-β on spheroids, we used a step-wise procedure as described below. We first collected A549 spheroids from an Elplasia 96-well plate after 4 days of culture duration. The collected spheroids were then transferred to an empty well of a separate Corning Ultra-Low Attachment (ULA) 96-well plate. Once all the spheroids settled at the bottom of the well after 5 minutes, the cell culture media was aspirated out leaving only the spheroids in the well. A small volume of 5 wt% gelMA was added to this well to make a hydrogel-spheroid suspension. The hydrogel-spheroid suspension was then pipetted into the middle channel of the microfluidic device allowing entry of multiple spheroids. Once the middle channel was full, we gently removed the pipette from the inlet without introducing any air bubbles. The chip was then transferred to a microscope stage for UV-crosslinking at 385 nm laser source for 45 seconds using a 5× objective lens. After UV-irradiation, the hydrogel undergoes irreversible chemical crosslinking and acts as a 3D scaffold for spheroids (Fig. 1B). To generate IF, we operated the microfluidic device as described in section 2.4. For experiments to study the effect of interstitial flow on A549 spheroids, the sample reservoir for the top channel was replaced with cell culture medium (DMEM, high glucose, 10% v/v FBS, 1% v/v Antibiotics). For IF with exogenous TGF-β experiments on A549 spheroids, we supplemented the culture medium with exogenous TGF-β (stock concentration; 5 μg mL^−1^) to achieve a final concentration of 0.1–10 ng mL^−1^. Bright-field and fluorescent images of the spheroids were captured on an inverted fluorescence microscope (Zeiss Axio-Observer) at an interval of 1 hour for a duration of 70 hours using a 20×/NA 0.16 air objective and ORCA Flash 4.0 V2 (Hamamatsu) digital camera with a resolution of 2048 × 2048 pixels. We used Software Autofocus strategy with the best contrast method to reduce background or out of focus fluorescence signal. For the GFP and RFP fluorescence, we used the 488 LED source (ex: 488 nm; emm: 520 nm) and 543 LED source (ex: 543 nm; emm: 590 nm), respectively. All experiments were conducted at 37 °C and 5% CO_2_ using a stage top incubator (ibidi). Bright-field images were taken at 10% light intensity and 100 millisecond exposure time. Fluorescence intensity for GFP and RFP images were analyzed *via* ImageJ (v1.53t, National Institute of Health, USA). A region of interest was created encircling the entire spheroid area for both GFP and RFP channel images, performed separately. This region of interest was quantified for pixel intensity density at every time point using measure function in ImageJ. The fluorescent intensity signal values were normalized with respect to the signal intensity at *t* = 0 h. CAGA-12-GFP and RFP reporter expression was plotted for multiple spheroids performed in 2 or 3 independent experiments. The device is robustly operational at pressure differences upto 30–35 mbar in the presence of spheroids. Increasing the pressure drop, resulted in the hydrogel structure breaking and interrupted uniform IF after a few hours. Within this pressure drop range, we were able to perform long-term culture experiments (up to 70 hours) to visualize cancer cell spheroid for fluorescence reporter signaling activity, and invasive response.

### 2.6 Microfluidic device setup for 2D cultured A549 cells under flow

Since A549 spheroids embedded in gelMA in a 3D-microfluidic chip cannot be retrieved to perform qPCR for target gene analyses, additional experiments were performed using dual-reporter A549 cells cultured in 2D-microfluidic without hydrogel matrix (see, Fig. S4†). These 2D-microfluidic experiments (without matrix) enabled us to extract A549 cells after the experiment to run qPCR analyses on TGF-β and EMT target genes to complement CAGA-12-GFP and VIM-RFP reporter expression quantification. Full experimental procedure is described in ESI† section 5. These studies additionally provide evidence of CAGA-12-GFP and VIMRFP reporter expression when A549 single cells were exposed to 2D-flow (without matrix) alone and in combination with exogenous TGF-β conditions, shown in Fig. S5(A and B).† Reporter expression was quantified in the following conditions: A) no-flow no-TGF-β (control), B) no-flow + TGF-β, C) flow − no-TGF-β, and D) flow + TGF-β conditions, shown in Fig. S5(C and D)† for CAGA-12-GFP and VIM-RFP reporter respectively.

### 2.7 qPCR analyses on target gene expression

Experiments performed using A549 cells in 2D-microfluidics (without matrix) in ESI† section 5, were further used to establish CAGA-12-GFP reporter activity with TGF-β target genes and confirm VIM-RFP expression with EMT target genes (full experimental procedure described in ESI† section 6.) We performed qPCR analyses on *CTGF*, *Serpin* (encoding PAI-1), and *Smad7* for TGF-β target genes and *E-cadherin*, *N-cadherin* and *Vimentin* for EMT target genes. Fig. S6 and S7† show the relative change in mRNA expression for each condition with respect to no-flow no TGF-β (control) for TGF-β and EMT target genes respectively. qPCR analyses further helped to establish the effect of 2D-flow and/or exogenous TGF-β induced reporter expression at a molecular level.

### 2.8 Statistical analysis

All statistical analysis was performed using Microsoft Excel (Microsoft Corporation, USA). The statistical significant differences between the two experimental groups were determined by Student *t*-test using the function *t-test: two samples with unequal variance* and *p* values below 0.05 were considered to be significant. We categorize statistical differences as following; *p* < 0.001 (***), *p* < 0.01 (**) and *p* < 0.05 (*).

## 3 Results and discussion

### 3.1 Exogenous TGF-β induced CAGA-12-GFP reporter response under interstitial flow conditions

**Fig. 2:**
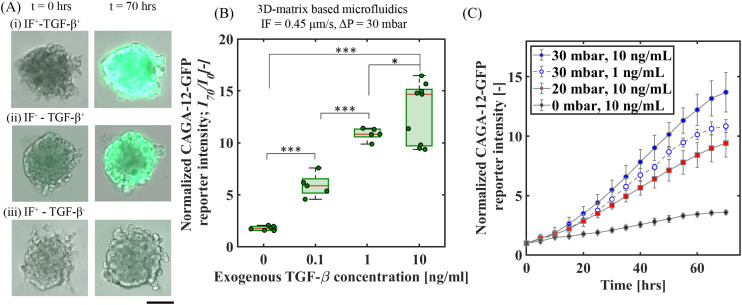
Exogenous TGF-β induced CAGA-12-GFP transcriptional reporter response of A549 spheroids under interstitial flow (IF) and no-flow conditions. (A) 20× GFP and bright-field merged microscope images of A549 spheroids at *t* = 0 and 70 h showing transcriptional-reporter intensity upregulation for the following conditions: (i) IF^+^–TGF-β^+^(10 ng mL^−1^), (ii) IF^−^–TGF-β^+^(10 ng mL^−1^) and (iii) IF^+^–TGF-β^−^, scale bar: 100 μm. (B): Quantitative measurement of normalized CAGA-12-GFP reporter signal intensity at *t* = 70 h for varying exogenous TGF-β concentrations under fixed IF. (C): Time series quantification of fluorescence signal intensity (with time intervals of 5 h) for CAGA-12-GFP upregulation profile under different IF (Δ*P* = 20 mbar and 30 mbar) and exogenous TGF-β conditions (1 and 10 ng mL^−1^), for *n* = 3 spheroids in each condition.

To analyze the effect of exogenous TGF-β under IF on Smad3/4-dependent transcriptional reporter response, we first examined the overall CAGA-12-GFP reporter fluorescence intensities at the end of 70 h for a fixed *C*_0_ = 10 ng ml^−1^ of exogenous TGF-β: (i) with IF (IF^+^TGF-β^+^) and (ii) without IF (IF^−^TGF-β^+^). These two conditions are contrasted with an IF condition without any exogenous TGF-β (IF^+^TGF-β^−^). Fig. 2A shows the bright-field images superposed with GFP fluorescence intensity at *t* = 0 and 70 h for these three conditions. The IF conditions were obtained under a fixed pressure gradient of Δ*P* = 30 mbar, equivalent of an average interstitial fluid velocity, *u*_m_ = 0.45 μm s^−1^ (measured separately *via* an independent experiment; see Fig. S3B†). We observed an enhanced CAGA-12-GFP reporter expression with the addition of exogenous TGF-β (2A(ii) *vs.* (iii)), which becomes further amplified across the spheroid under the imposed IF (2A(i) *vs.* (ii)). This observation strongly suggests that IF enhances the exogenous TGF-β induced Smad-signaling activity in A549 spheroids.

The statistics of relative increase in the CAGA-12-GFP reporter expression (*I*_70_) at *t* = 70 h for these conditions were quantified for multiple spheroids based on the intensity readouts normalized by baseline values (*I*_0_) at *t* = 0 h. The box plot in Fig. 2B shows the average reporter signal intensity (*I*_70_/*I*_0_) as a function of varying exogenous TGF-β conditions under fixed IF at Δ*P* = 30 mbar. Among all the reported conditions, we observed the strongest reporter upregulation (*I*_70_/*I*_0_ = 13 ± 2.73) for an exogenous TGF-β concentration of *C*_0_ = 10 ng mL^−1^ under IF (*i.e.* IF^+^–TGF-β^+^ (10 ng mL^−1^)). We also observed that supplying exogenous TGF-β (10 ng mL^−1^) without IF (IF^−^–TGF-β (10 ng mL^−1^)) has approximately 73% lower reporter expression when compared to IF^+^TGF-β^+^ (10 ng mL^−1^), see Fig. S8(A).† This result highlights a potentiating effect of IF towards an enhanced exogenous TGF-β induced Smad-signaling activity measured *via* upregulation in CAGA-12-GFP transcriptional reporter response. In addition, we studied the effect of different IF velocities without exogenous TGF-β supplement for Smad-dependent CAGA-12-GFP reporter activity. We observed minimal reporter gene upregulation at *t* = 70 h which can be linked to the inactivity of the Smad-pathway in the absence of exogenous TGF-β, see Fig. S9(A and C).†

To explore this potentiating effect between IF and exogenous TGF-β further, we employed time-lapse imaging to monitor the CAGA-12-GFP reporter expression profile through 70 h under varying IF (*u*_m_ = 0.2 μm s^−1^ and 0.45 μm s^−1^ at Δ*P* = 20 and 30 mbar, respectively) and exogenous TGF-β concentrations (*C*_0_ = 1 and 10 ng mL^−1^). Fig. 2C shows the time-wise variations in 〈*I*/*I*_0_〉 for the following three combinations of IF and exogenous TGF-β concentrations – IF^+^TGF-β^+^: Δ*P* = 30 mbar; *C*_0_ = 10 ng ml^−1^, Δ*P* = 20 mbar; *C*_0_ = 10 ng ml^−1^, compared with the Δ*P* = 0 mbar; *C*_0_ = 10 ng ml^−1^*i.e.* no-IF condition. We observed a clear influence on the CAGA-12-GFP signal intensity profile with changing IF pressure gradients. For *C*_0_ = 10 ng ml^−1^, the IF condition at Δ*P* = 30 mbar resulted in the fastest non-linear increase in the fluorescence signal intensity profile that begins to show saturation over the 70 hour time period (Fig. 2C). For the IF at Δ*P* = 20 mbar and the no-IF conditions, fluorescence signal intensity showed relatively slower upregulation responses (Fig. 2C). Interestingly, for the IF condition of Δ*P* = 30 mbar, decreasing the exogenous TGF-β concentration by an order of magnitude (*i.e. C*_0_ = 1 ng ml^−1^) still resulted in an upregulation response faster than the Δ*P* = 20 mbar; *C*_0_ = 10 ng ml^−1^ condition. This observation indicates that the Smad-dependent transcriptional reporter response is weakly sensitive to the exogenous TGF-β concentration, but shows a strong dependence on the IF. Under the fixed Δ*P* = 30 mbar, for both *C*_0_ = 1 and 10 ng ml^−1^ the upregulation rates are nearly equal between 25–55 h, after an initial delayed response for the former. Additionally, we performed similar experiments under no-IF conditions with exogenous TGF-β concentration (1 and 10 ng mL^−1^). The CAGA-12-GFP expression for 1 ng mL^−1^ and 10 ng mL^−1^ under no-IF showed a similar upregulation profile (see Fig. S13†), suggesting that the transcriptional response is fairly independent of the exogenous TGF-β concentration greater than *C*_0_ = 1 ng ml^−1^. To follow-up on the potentiating effect of IF in our 3D-microfluidic A549 spheroid experiments, we performed additional experiments in a 2D-microfluidic system (without matrix) using dual-reporter A549 cells (refer to ESI† section 5 for detailed experimental procedure). In Fig. S5C,† dual-reporter A549 cells showed maximum CAGA-12-GFP reporter expression in the presence of 2D-flow and exogenous TGF-β condition. The reporter response is similar to the observations in 3D-matrix microfluidic experiments with A549 spheroids displaying maximum activity under IF and exogenous TGF-β condition (Fig. 2B). In addition, in 2D-microfluidic experiments without matrix, A549 cells showed only a modest upregulation in CAGA-12-GFP reporter expression under 2D-flow conditions without exogenous TGF-β when compared to control condition with no-flow and no TGF-β, see Fig. S5C.† This observation indicates that biophysical forces arising from 2D-flow induced fluid-shear stress feeds into the Smad-pathway that leads to CAGA-12-GFP transcriptional reporter activity. Furthermore, qPCR target gene analysis was performed on A549 cells exposed to 2D-flow and/or exogenous TGF-β conditions (refer to ESI† section 6 for full experimental procedure) to confirm for Smad-dependent CAGA-12-GFP transcriptional reporter activity with change in TGF-β target gene expression. Maximum target gene expression for *CTGF*, *Serpin1 encoding PAI-1*, and *Smad7* was observed in A549 cells stimulated with 2D-flow and exogenous TGF-β conditions, see Fig. S6.† TGF-β target gene expression for cells exposed to only 2D-flow (no exogenous TGF-β) showed an increase in *Serpin1* and *Smad7* target genes. This further highlights that flow alone activates Smad-dependent CAGA-12-GFP reporter response.

It has been proposed that the transcriptional gene response from Smad-signaling pathway is dependent on a chain of reaction kinetics initiated with binding of exogenous TGF-β molecules at the active receptor sites.^49^ These reaction kinetics include expression level of TGF-β receptors and Smads and its activation state, ability to translocate into the nucleus, ability to interact with other transcription factors, co-activators, co-repressors, and chromatin modulators *etc.*^49^ The local concentration of available TGF-β should influence the conversion capacity of available receptor sites to activated receptor sites upon successful binding. Additionally, active TGF-β availability is tightly controlled by its interaction with ECM proteins and its ability to present itself to signaling receptors is regulated by co-receptors (without intrinsic enzymatic motif), by integrins and other receptor molecules.^25^ The upregulation rate of the transcriptional gene response is controlled by the density of receptor sites (*i.e.* number of available sites) and the reaction rate constant. To validate the TGF-β receptor binding affinity and activation in response to exogenous TGF-β molecules, we used a well-known TGF-β type I receptor inhibitor (SB-431542). This small molecule inhibitor is used to inhibit all TGF-β type I receptor kinase activity. We first performed experiments in 3D-static (without IF) conditions with A549 spheroids embedded in 5 wt% gelMA with and without SB-431542 inhibitor treatment; followed by stimulation with exogenous TGF-β (10 ng mL^−1^), full experimental procedure is described in ESI† section 9(i). Fig. S10† shows A549 spheroids stimulated with only exogenous TGF-β showed Smad-dependent CAGA-12-GFP transcriptional activity. On the other hand, A549 spheroids initially treated with SB-431542 (10 μM) inhibitor and subsequently stimulated with exogenous TGF-β (10 ng mL^−1^) showed no CAGA-12-GFP reporter activity. This is a result of receptor kinase activity inhibition. In addition, we saw similar effect of SB-431542 inhibitor treatment on CAGA-12-GFP and VIM-RFP reporter expression in 2D-microfluidic experiments on dual-reporter A549 single cells (without matrix) shown in Fig. S11 and S12† (full experimental procedure described in ESI† section 9(ii) and (iii)). With these experiments we concluded that, CAGA-12-GFP transcriptional reporter activity is Smad-dependent, which is a result of TGF-β receptor activation upon binding with TGF-β molecules. In our 3D microfluidic studies, we estimated the evolution of local TGF-β concentration in the vicinity of a spheroid. We performed 2D mass transport simulations using the finite-element method (implemented in COMSOL Multiphysics) by varying the IF conditions and the input concentration of exogenous TGF-β (Fig. S13A†). At 350–400 minute mark, each condition has achieved its respective saturation concentration (*C*_0_) of the exogenous TGF-β (Fig. S13A†). Additionally, we tested the penetration of exogenous TGF-β in the presence of IF and no-IF. To do this, we used FITC-labelled dextran (20 kDa) tracer particles (similar to TGF-β molecule, 25 kDa).

We observed that IF enhanced the penetration of FITC-dextran particles quantified by the increase in fluorescence intensity at pressure difference of 20 and 30 mbar, compared to 0 mbar, see Fig. S13B.† Although IF can increase the penetration of exogenous TGF-β molecules, extrapolating from results obtained with dextran tracer particles, this effect appears moderate (a factor of 2) compared to the fold change due to physical forces (such as shear and compressive stress). Since the upregulation of CAGA-12-GFP was found to be fairly independent of exogenous TGF-β concentration even under no-IF condition (see Fig. S14†), we expect that all active binding sites on the spheroid interface are activated for each case by this 250–400 minute mark of the experiment. Comparing this analysis with the results in Fig. 2C suggests that besides the exogenous TGF-β, there are additional biophysical forces induced mechanotransduction pathways that influence an enhanced CAGA-12-GFP reporter upregulation from TGF-β induced Smad-signaling activity.

Cancer cells have the ability to respond to mechanical cues (matrix stiffness, fluid shear stress and compressive forces) by activation of cell surface mechanosensors such as integrins, focal adhesion complex, transient receptor potential (TRP) ion channels and YAP/TAZ signaling pathway.^23,50–53^ Activation of mechanotransduction signaling pathways may lead to transcriptional activity of YAP/TAZ, commonly identified to promote cancer cell invasion and trigger EMT signaling pathways.^22,54,55^ Earlier studies have linked mechanotransduction induced EMT for cancer cells under a flow-induced shear stress of 0.1–3 Pa.^56–58^ These studies were performed on 2D monolayer culture without an extracellular matrix environment. The shear stress induced by an interstitial fluid flow is typically reported to be in the order of 0.01 Pa.^59^ These values are reported for cells cultured on 2D substrate subjected to IF velocities in microfluidic systems. In our 3D-matrix based microfluidic study, we found that IF generated *via* a pressure gradient leads to both flow-induced shear stress and compressive stress contributing to the Smad-dependent transcriptional reporter activity. Based on our simulation, the shear stress on a 2D spheroid model interface embedded in a low permeability matrix (mimicking the properties of gelMA used in the experiments) was found to be relatively low (∼0.1–0.3 mPa, see Fig. S3C†). The compressive/normal stress caused by hydrodynamic pressure at the spheroid interface is significantly high (∼1 and 2 kPa, see Fig. S3D†). Previous literature has highlighted the role of matrix stiffness and matrix-induced compressive forces activating key mechanotransduction pathways (Wnt, Hippo, PI3-AKT, TGF-β) for cancer cell proliferation and migration.^60–62^ Since our 3D-microfluidic platform does not allow to quickly access A549 spheroids embedded in hydrogel matrix, we are unable to perform qPCR analyses to confirm for specific mechanotransduction pathway underplay. The long time it takes to isolate the spheroids from the device is likely to affect the gene expression profile within the spheroid. A detailed study to identify specific mechanotransduction pathways under IF upregulated in a 3D-TME exposed to IF will shed more light on this hypothesis.

### 3.2 Local fluorescence profile of CAGA-12-GFP reporter activity in A549 spheroid under varying IF-exogenous TGF-β condition

**Fig. 3:**
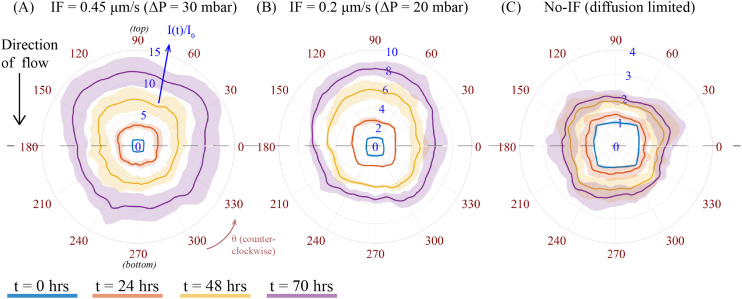
CAGA-12-GFP reporter fluorescence profile under a fixed exogenous TGF-β concentration (10 ng mL^−1^) and varying IF and no-flow conditions. Polar plot of radially averaged fluorescence intensity is denoted in *I*(*t*)/*I*_0_ (in blue) at different time intervals (represented with solid lines of different color). The solid line and the shaded region of a particular color of a particular time point show averaged and standard deviation of fluorescence intensity respectively for *n* = 3 spheroids. The evolution of fluorescence intensity at the top and bottom half of the spheroid (from the dashed line) shows the CAGA-12-GFP reporter expression profile for conditions: (A) IF^+^(0.45 μm s^−1^)TGF-β^+^, (B) IF^+^(0.2 μm s^−1^)TGF-β^+^, and (C) no flow, IF^−^TGF-β^+^.

To examine the local fluorescence profile of Smad-dependent CAGA-12-GFP transcriptional reporter activity in a spheroid as a consequence of varying IF from different pressure gradient, we compared evolution of fluorescence intensity at different times for a fixed exogenous TGF-β concentration (*C*_0_ = 10 ng mL^−1^) under two different values of IF (Δ*P* = 20 and 30 mbar) and no-IF conditions. To represent the local heterogeneity in fluorescence intensity of a spheroid exposed to varying IF, we used the polar transformer function in ImageJ. An example of methodology for this analysis technique on one set of spheroid images at different time intervals is shown in ESI† section 12. This image analysis function converts a 2D-microscope image from a Cartesian coordinate system to a polar coordinate system (*r*, *θ*), see Fig. S15(A and B).† Using this function, we then measured the radially averaged intensity of a polar coordinate (*r*, *θ*) transformed image at different time intervals (*t* = 0, 24, 48 and 70 h) of a particular spheroid, see Fig. S15(B).† We then plot the radially averaged intensity on the azimuthal scale, *i.e. θ* = 0 to 360 degrees, see Fig. S15(C),† which constructs the evolution of fluorescence intensity profile corresponding to the spheroid fluorescence intensity at different time intervals. The intensity profile for each spheroid was normalized to its initial fluorescence value at *t* = 0 h. The difference in intensity of fluorescence signal among these conditions are influenced by the varying IF conditions (as previously discussed in section 3.1, Fig. 2C). Fig. 3 compares the intensity profiles of spheroids under varying IF (Fig. 3A and B) and no-IF condition (Fig. 3C). The fluorescence intensity (*I*(*t*)/*I*_0_) is plotted on the scale between 0 and 15 (represented in blue). Averaged fluorescence intensity at each time point (denoted with different colors) is represented with a solid line and standard deviation in intensity with its corresponding shaded region. The azimuthal axis, *θ* (counterclockwise, in red) is used to represent the local fluorescence profile of the spheroid. When spheroids are exposed to IF, we can observe the asymmetry by the averaged fluorescence intensity profile of spheroids (*n* = 3) at *θ* = 90 (top) and 270 (bottom) degrees, shown in Fig. 3. In Fig. 3A, spheroids under IF at Δ*P* = 30 mbar, show an average fluorescence intensity at the top of the spheroid (*θ* = 90 degree) is 12.3 ± 2.65 and at the bottom (*θ* = 270 degree) is 9.8 ± 2.17 at *t* = 70 h (in purple). We observed a similar trend in heterogeneity of fluorescence intensity for spheroids under IF at Δ*P* = 20 mbar (Fig. 3B). Fig. 3C shows that the fluorescence intensity profiles of spheroids under no-IF condition shows axisymmetry, *i.e.* no noticeable change in fluorescence intensity at the top/bottom of the spheroid. In no-IF condition, fluorescence intensity is not influenced by any hydrodynamic effect or fluid induced shear/compressive stress. We suspect that the absence of biomechanical stress (IF) and inactivation of mechanotransduction pathways justifies axisymmetric fluorescence profiles in only exogenous TGF-β exposed A549 spheroids. The top-bottom asymmetry in CAGA-12-GFP upregulation profiles along the direction of flow (top to bottom) is a result of the applied IF conditions originating from varying hydrodynamic pressure.

### 3.3 Exogenous TGF-β induced vimentin activity towards cancer cell motility exposed to interstitial flow in A549 spheroids

**Fig. 4:**
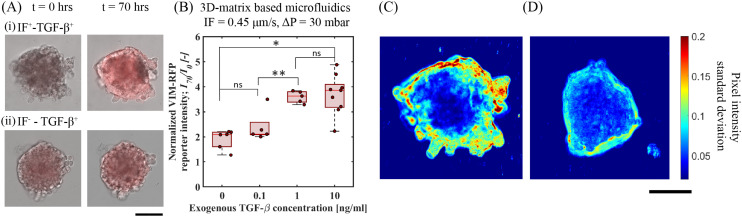
Upregulation in vimentin expression as measured *via* VIM-RFP reporter gene response toward cell motility in A549 spheroids. (A) 20× RFP and bright-field channel merged microscope images of A549 spheroids at *t* = 0 and 70 h showing gene-reporter intensity upregulation in the following conditions. (i) IF^+^–TGF-β^+^(10 ng mL^−1^) and (ii) IF^−^–TGF-β^+^(10 ng mL^−1^), scale bar: 100 μm. (B) Quantitative measurement of normalized VIM-RFP reporter signal intensity at *t* = 70 h for varying exogenous TGF-β under fixed IF (0.45 μm s^−1^) at Δ*P* = 30 mbar. (C) and (D) Standard deviation analysis showing cellular motion activity at the periphery of A549 spheroids under IF and no-flow conditions with exogenous TGF-β (10 ng mL^−1^), scale bar: 100 μm.

To further explore the potentiating effect of IF with exogenous TGF-β, we examined the upregulation of vimentin as measured by determining the VIM-RFP reporter response. Vimentin, a key mesenchymal biomarker, is upregulated in lung cancer cells in the presence of TGF-β towards EMT response.^29,31,32^ We measured the upregulation in vimentin expression activity by quantifying VIM-RFP reporter expression under IF and no-flow conditions in the presence of exogenous TGF-β. Fig. 4A shows the superposed microscope images of bright-field and RFP channels at *t* = 0 and 70 h. We observed an enhanced VIM-RFP reporter expression with IF and exogenous TGF-β (IF^+^TGF-β^+^) (Fig. 4A(i)) compared to IF^−^TGF-β^+^ (Fig. 4A(ii)) (no flow condition). Fig. 4B shows the quantified VIM-RFP signal upregulation for the same conditions (described in section 3.1). We observed that the strongest reporter upregulation (*I*_70_/*I*_0_ = 3.7 ± 0.74) with an exogenous TGF-β concentration of 10 ng mL^−1^ under IF (Δ*P* = 30 mbar) (*i.e.* IF^+^–TGF-β^+^(10 ng mL^−1^)). We also observed that the upregulation of VIM-RFP intensities for TGF-β concentration 10 ng mL^−1^ and 1 ng mL^−1^ (*I*_70_/*I*_0_ = 3.6 ± 0.2) under IF (Fig. 2B) showed no significant difference. Moreover, the VIM-RFP expression induced by exogenous TGF-β (10 ng mL^−1^) under no-flow (*I*_70_/*I*_0_ = 1.41 ± 0.08) is 62% lower compared to spheroids under IF with exogenous TGF-β (10 ng mL^−1^), refer Fig. S8(B).† In addition, we studied the effect of varying IF without exogenous TGF-β for VIM-RFP reporter response. We observed no change in reporter activity at *t* = 70 h in A549 spheroids as a result of varying IF conditions, see Fig. S9(B and C).† Following up with 2D-microfluidic experiments, we observed maximum VIM-RFP expression for 2D-flow and exogenous TGF-β conditions, similar to observations made in 3D-microfluidics in A549 spheroids. In addition, A549 cells exposed to 2D-flow (without matrix) showed upregulation in VIM-RFP reporter expression when compared to no-flow no TGF-β (control) condition, see Fig. S5(B and D).† Therefore, we propose that the IF in the presence of exogenous TGF-β has a potentiating effect that is further responsible for producing an increased VIM-RFP reporter expression corresponding to a higher vimentin abundance. These results highlight the potential involvement of mechanotransduction induced signaling pathways that contribute towards upregulation in mesenchymal marker in A549 cells.

We quantified spheroid peripheral activity for increased cellular motion activity. The cellular motion activity here is referred to as cells at the edge of a spheroid that responds to biophysical and biochemical cues characterized with an increased motility. When stimulated with exogenous TGF-β and IF, we observed an increase VIM-RFP reporter expression corresponding to increase in vimentin abundance protein. This is identified as a mesenchymal biomarker, that is often associated with a phenotype observed in motile cancer cells. To quantify cellular motion activity, we performed temporal standard-deviation analysis of bright-field time-lapse images. This analysis technique detected the change in pixel intensity value at each time point for the duration of the entire experiment. After processing all images, the final image represents the qualitative measurement of the standard deviation change in pixel value corresponding to the cellular motion activity at the spheroid periphery. Fig. 4C and D demonstrates a comparison of an A549 spheroid under IF (0.45 μm s^−1^, Δ*P* = 30 mbar) and no-IF both stimulated with exogenous TGF-β (10 ng mL^−1^). Refer to ESI† Movie S1 and S2 (corresponding to spheroid in Fig. 4C and D respectively) for time lapse video of cellular motion at spheroid edges embedded in gelMA matrix. In Fig. 4C, the A549 lung tumor spheroid shows increased cellular motion activity under IF^+^–TGF-β^+^(10 ng mL^−1^) condition with a larger standard deviation measured correlating with increased cellular motion activity. The increase in cell cellular motion activity was mostly observed at the top/side section of the spheroid periphery. From Fig. 4D, only exogenous TGF-β is insufficient to produce cellular motion activity depicted with low standard deviation of pixel value change. In these conditions, the A549 spheroid periphery did not show active cellular motion (refer to ESI† Movie, S2). The increased activity in the presence of IF and exogenous TGF-β condition can be linked to the hypothesis of mechanotransduction pathway induced activity. Additional experiments performed in 2D-microfluidics for qPCR analyses (refer to ESI† section 6, Fig. S7) showed that dual-reporter A549 cells exposed to 2D-flow (without matrix) led to downregulation in *E-cadherin* expression and upregulation in *N-cadherin* and *Vimentin* characteristic of EMT target genes. This brings us closer to our hypothesis of normal/shear stress activating mechanosensors for triggering additional mechanotransduction signaling pathways. These findings highlight the importance of IF and exogenous TGF-β that directly influence A549 tumor cells to undergo active cellular motion in a tumor microenvironment.

## 4 Conclusions and outlook

We used a 3D-matrix based microfluidic platform to investigate the potentiating effect of IF on exogenous TGF-β induced Smad-signaling activity in A549 lung cancer spheroids. Our platform allowed us to embed cancer spheroids in 3D using gelMA hydrogel as a relevant ECM material. This integrated platform of porous hydrogel material and cancer spheroid allowed us to mimic IF conditions experienced by a tumor in a TME. One advantage of this microfluidic platform was the ability to investigate cancer cell–matrix interactions over time, allowing us to observe the effects of varying biophysical conditions and biochemical signals. By studying the interplay between biophysical components (hydrogel matrix and IF), and the externally introduced cytokine (exogenous TGF-β), we aimed to better understand how these factors contribute to cancer spheroid response and invasive behavior. To this end, we monitored the upregulation in transcriptional reporter response (CAGA-12-GFP) and vimentin abundance protein (VIM-RFP reporter) in A549 lung spheroids using real-time imaging of artificial gene reporter constructs. Our findings suggest that the addition of IF within the 3D-matrix significantly enhances the CAGA-12-GFP reporter response from Smad-signaling activities upregulated by exogenous TGF-β. This also leads to an increase in the abundance of vimentin protein measured *via* upregulation in VIM-RFP reporter expression and increased cellular motion activity observed at the spheroid periphery in a matrix microenvironment. Additional experiments performed in 2D-microfluidic system (without matrix) further confirmed the mRNA expression of TGF-β and EMT target genes to establish CAGA-12-GFP and VIM-RFP reporter functionality of the dual-reporter A549 cells. In 2D-microfluidic experiments (without matrix), flow (without exogenous TGF-β) alone has a clear evidence of upregulation in Smad-dependent CAGA-12-GFP reporter activity and *vimentin* gene with qPCR analyses. Moreover, 2D-flow also resulted in downregulation of *E-cadherin*. The fluorescent reporter activity confirmed by target gene analyses indicates that 2D-flow alone activates parallel pathways that feed into the Smad pathway leading to Smad-induced transcriptional reporter activity. In 2D-microfluidic (without matrix) and 3D-microfluidic (with matrix), CAGA-12-GFP and VIM-RFP showed maximum reporter intensity when exposed to flow and exogenous TGF-β. However, in 3D-microfluidic experiments, A549 spheroids embedded in gelMA showed no clear upregulation in reporter expression when exposed to only IF (without exogenous TGF-β), in contrast to 2D-microfluidic experiments. The reporter expression was potentiated by IF only in the presence of exogenous TGF-β.

The reporter expression showed clear dependency on the magnitude of IF applied (increasing pressure gradient) and showed less sensitivity to exogenous TGF-β concentration. Using complimentary numerical simulation on a 2D spheroid model, we further characterized the mass transport of TGF-β, flow induced shear and normal stresses on the spheroid interface under different IF conditions. Based on these results and qPCR analyses, we hypothesize that exogenous TGF-β induced Smad-signaling and vimentin expression is further upregulated from potentiating effect of interstitial flow mediated mechanotransduction pathways.

The 3D microfluidic platform introduced in this study has the potential to expand beyond tumor spheroid models, and can be applied to heterogeneous tumor spheroid, stromal cells, cancer-associated fibroblasts (CAFs) and immune cells. This versatility brings us closer to mimicking *in vivo* tumor microenvironment (TME) conditions.

## Author contributions

Z. R., P. T. D. and P. E. B. conceived the ideas and designed the experiments. Z. R. carried out the experiments and collected the data. H. R. and M. T. carried out additional experiments on interstitial flow rate measurement and rheology of the hydrogel. G. V. D. Z. performed qPCR analyses for all experiments. Z. R. and A. B. performed data and image analysis. A. B. performed numerical simulations and developed MATLAB codes for image analysis. Z. R. wrote the paper and A. B., V. G., P. T. D. and P. E. B. edited it.

## Conflicts of interest

There are no conflicts to declare.

## Supplementary Material

LC-024-D3LC00886J-s001

LC-024-D3LC00886J-s002

LC-024-D3LC00886J-s003
