# Supplementary material for: Interstitial flow potentiates TGF-β/Smad-signaling activity in lung cancer spheroids in a 3D-microfluidic chip
Source: Lab Chip. 2023 Dec 4;24(3):422–33. doi: 10.1039/d3lc00886j (PMC10826459; doi:10.1039/d3lc00886j)
Supplement: LC-024-D3LC00886J-s001 [file LC-024-D3LC00886J-s001.pdf]

Electronic Supplementary Material (ESI) for Lab on a Chip.
This journal is © The Royal Society of Chemistry 2023

Interstitial flow potentiates TGF-β/Smad-signaling activity in lung cancer spheroids in a 3D-microfluidic chip – Supplementary Material

Zaid Rahman,[a] Ankur Deep Bordoloi,[a] Haifa Rouhana,[a] Margherita Tavasso,[a] Gerard van der Zon,[b] Valeria Garbin,[a] Peter ten Dijke,[b] and Pouyan E. Boukany *[a]

[a] Department of Chemical Engineering, Delft University of Technology, Delft, The Netherlands.

[b] Department of Cell and Chemical Biology and Oncode Institute, Leiden University Medical Center, Leiden, The Netherlands

*Corresponding author

Electronic Supplementary Information (ESI)

1. Storage Modulus and stiffness characterization

The viscoelastic properties of GelMA were investigated with a modular rotational rheometer (DSR 502, Anton Paar) equipped with a parallel plate of diameter of 25 mm. A volume of 500 µL of gel was crosslinked on a glass slide in the same conditions as in the microfluidic device. Frequency sweeps were performed from low to high frequencies and vice-versa (0.1 to 100 rad/s at a fixed strain of 1%) at room temperature (22^{0}C). The results are similar as the measurements occur within the linear viscoelastic (LVE) regime of the gel.

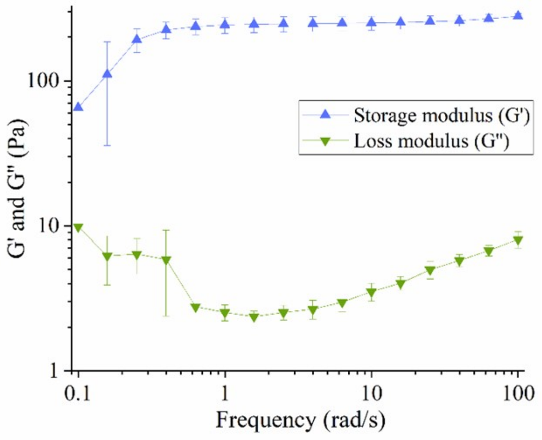

Figure S1: Frequency dependence of storage modulus (G') and loss modulus (G'') of 5 wt.% gelMA in small-amplitude oscillatory shear measurement (at a fixed strain amplitude of 1.0% at room temperature).

2. Design and fabrication of 3-D microfluidic chip

The master wafer was fabricated by a standard photo-lithography technique using a µMLA laserwriter (Heidelberg Instruments) on a 4-inch silicon wafer at the Kavli Nanolab Delft. The top and the bottom channel are fluidic channels and the middle channel is separated via triangular shaped pillars where spheroids are suspended in a hydrogel. The hydrogel is confined in the middle channel primarily due to the presence of evenly spaced triangular posts[1,2]. We optimized the photolithography procedure to achieve a final channel height of 280 ± 25 µm to embed cancer spheroids in 3D. To obtain this, we first spin-coated SU-8 2150 (acquired from Kayaku Advanced Materials) negative photoresist in two steps. First step was at 500 rpm for 10 seconds with an acceleration of 100 rpm per second followed by 2300 rpm for

30 seconds with an acceleration of 300 rpm per second. The silicon-wafer with SU-8 was then transferred to a hot plate to soft-bake at 65^{0}C for 15 minutes, followed by 95^{0}C for 90 minutes. After soft-bake, the wafer was loaded onto the laserwriter sample holder stage. The AutoCAD design was uploaded to the laserwriter software to print the design using a 365 nm laser source. The wafer was post baked at 65^{0}C for 15 minutes, followed by 95^{0}C for 5 minutes and developed in SU- 8 photo-resist developer (mr-Dev 600, Micro resist technology). The microfeatures are constantly checked under an optical microscope to adjust the photo-resist developing time period in order to avoid over or under-development. The height of the channels was determined by a Dektak profilometer. The profilometer measures channel height 280 µm ± 25 µm. The master wafer was coated with trichloro(1H,1H,2H,2H-perfluorooctyl)silane to create a hydrophobic surface for easy demolding. Polydimethylsiloxane (PDMS) based microfluidic chips were prepared using the mixture of Sylgard 184 and curing agent (at a ratio of 10:1). Individual chips were then cut out and inlets/outlets were punched in the PDMS slab consisting of the design to insert tubings (PTFE, inner diameter: 0.8mm, outer diameter: 1.6mm) for fluidic-channel operation. Final bonding is performed by plasma cleaning (Harrick Plasma) of the PDMS slab and a glass coverslip (#1.5) for two 2 minutes 20 seconds to facilitate final bonding. Finally, the assembled device was kept in the oven at 65^{0}C to bond overnight and restore the hydrophobicity of the channels. Afterwards, the microfluidic devices can be stored indefinitely and sterilized by UV-irradiation for 20 minutes before the start of each experiment.

3. Hydraulic permeability (K) measurement to compute interstitial flow velocity

To quantify interstitial flow velocity through 5 wt.% gelMA hydrogel, we first identified the hydraulic permeability (K) of the hydrogel. We used Rhodamine B (Merck Sigma) as tracer particle and measured the distance covered by the fluorescent dye through gelMA in a specific time frame. These studies are performed only with the hydrogel material and no spheroids. The microfluidic chip was prepared by first filling the hydrogel channel with 5wt.% gelMA, followed by crosslinking using 5x-obective lens with a LED UV-light source (385 nm) from Colibri Inverted microscope for 45 seconds. We prepared the dye solution by mixing a 1% (v/v) solution of Rhodamine B in 1X Dulbecco's Phosphate Buffer Solution (DPBS, Sigma Aldrich). This dye solution is used as a fluid reservoir connected to the Fluigent MFCS-EZ pressure pump to allow flow into the microfluidic channels. The tubings (inner diameter: 0.8 mm and outer diameter: 1.6 mm) from the reservoir are connected to the inlets (P_1) of the top channel. The bottom channel inlets (P_2) were connected to an empty reservoir. By applying a pressure gradient, we generated a flow through the porous hydrogel material, see Fig. S2 (for experimental setup). To validate IF, we tracked fluorescent dye travel with time-lapse imaging at an interval of 30 seconds. Images were taken on Zeiss Axio Observer Colibri 7 equipped with ORCA Flash 4.0 V2 (Hamamatsu) digital camera with a resolution of 2048 × 2048 pixels using 543nm LED laser source (excitation/emission: 543 nm/568nm), at 30% intensity laser and 1.58 seconds exposure time, with objective lens set to 5x. Flow velocity was then calculated using ImageJ for pressure gradients of ΔP: 20 mbar and 30 mbar. These pressure gradients were optimal since a high pressure gradient (> Δ40 mbar) disturbs the mechanical stability of the hydrogel and disrupts uniform interstitial flow. Images are exported from Zeiss software as .tif files and imported as a stack into ImageJ for analysis. The images were then straightened and converted into 8-bit color images. A vertical line was drawn as area of interest to plot profile of gray value vs. distance. Mean gray value of zero corresponds to

the last position of the dye at a specific time, this position was recorded for consecutive images for an average of 10 time points. The velocity was then calculated by dividing the distance traveled by the duration (between two consecutive images) and the average velocity on the line of interest is obtained. . The average velocity for ΔP = 20 mbar and 30 mbar is calculated at 0. 46 ± 0.21 µm/s and 0.72 ± 0.16 µm/s respectively (see Table 1 for average velocity measurements). All known parameters were plugged into Darcy's law (eq. 1) to obtain the hydraulic permeability (K) of the hydrogel.

$$u_m = \frac{\Delta P \times K}{L \times \mu} \quad \text{(eq.1)}$$

Where u_m is the fluid velocity across the porous network (m/s), ΔP is the pressure gradient across the porous network (Pa), L is the length of the porous network in the direction of the flow (m), K is the hydraulic permeability (m^2), and μ is the fluid dynamic viscosity (Pa s). The average permeability constants were measured at approximately 1.35x 10^{-16} m^2 for 5wt.% gelMA. The permeability of 5wt.% gelMA was compared to the values found in literature to be of approximately 0.1 μm^2/Pa.s for a high degree substitution 5wt.% gelMA[3], equivalent to 10^{-16} m^2.

Time (sec)	ΔP = 30 mbar		ΔP = 20 mbar	
	Distance travelled (µm)	Calculated average velocity, u_m (µm/s)	Distance travelled (µm)	Calculated average velocity, u_m (µm/s)
0	0	0		
30	23.97	0.79	8.20	0.27
60	32.18	1.07	21.54	0.71
90	24.61	0.82	18.29	0.61
120	18.23	0.60	10.09	0.33
150	15.14	0.50	22.71	0.75
180	22.72	0.75	5.04	0.16
210	22.08	0.73	12.62	0.42
240	22.08	0.73	20.82	0.69
270	14.51	0.48	14.51	0.48
300	23.35	0.77	4.41	0.14
	Average velocity	0.72	Average velocity	0.45
	Standard deviation	0.16	Standard deviation	0.26

Table S1: Average velocity measurement for ΔP = 20 and 30 mbar.

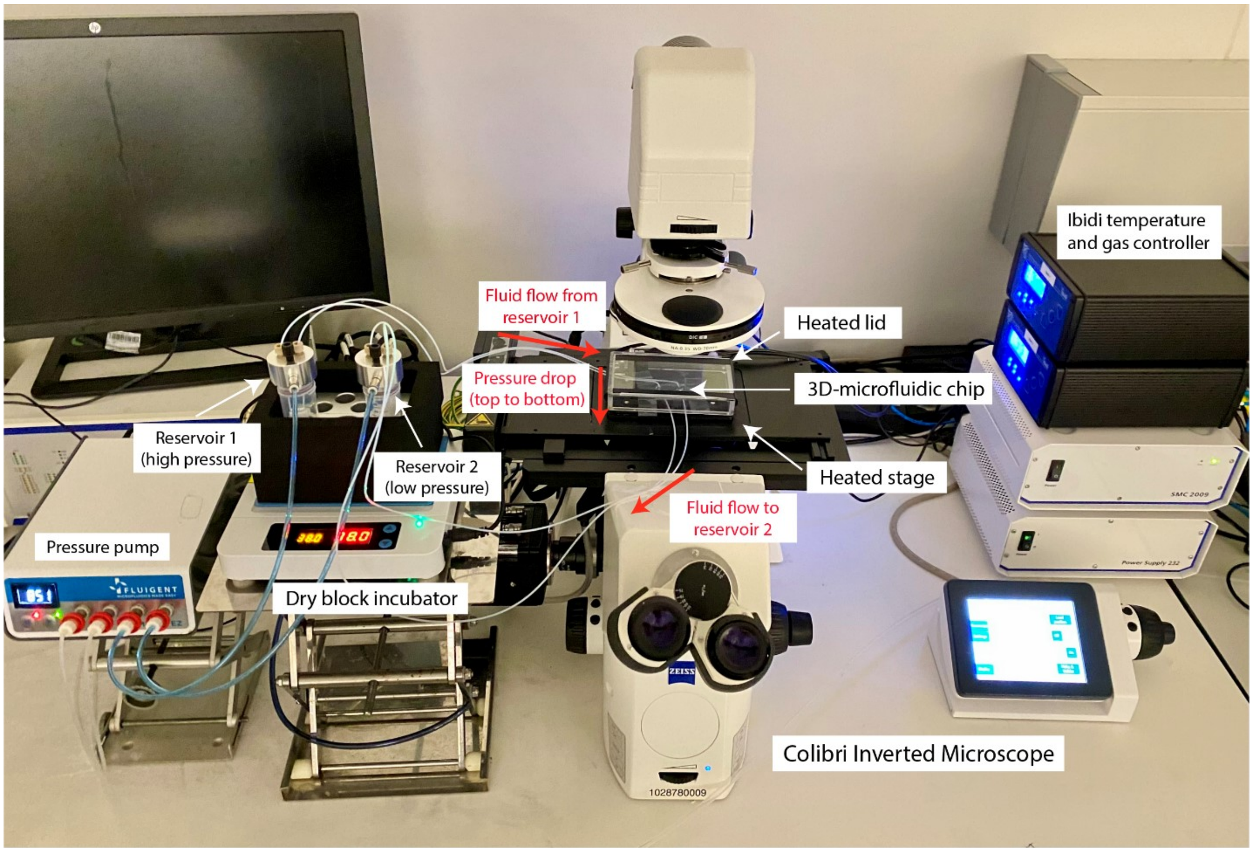

Figure S2: Experimental setup to generate interstitial flow in a 3D-microfluidic chip using a pressure pump. The reservoirs are kept in a dry block incubator maintained at 37^0C. The pressure pump channels are connected to the media reservoirs set at high and low pressure values to create a pressure gradient. The microfluidic chip is placed in a Ibidi stage top incubator equipped with a heated lid and a heated stage. The Ibidi temperature and gas controller box is used to maintain cell culture conditions at 37^{0-}C, 95% humidity and 5% CO_2. Colibri inverted microscope used for fluorescence time-lapse imaging.

4. Fluid velocity and stress profiles around the spheroid

To compute the interstitial flow velocity in the microfluidic model, we solved 2D incompressible Stoke's flow equations using inbuilt Free and Porous Media Flow interface in COMSOL Multiphysics. For simplicity, the spheroid was modeled as a solid circular region with a rigid boundary. A pressure gradient across the porous matrix was generated by imposing pressure P_1 at the inlets (A and B) and P_2 ($<P_1$) at the outlets (C and D). The velocity and pressure fields were computed over the domain discretized using a physics based unstructured mesh with approximately 3×10^5 elements with a minimum size of 1 µm, represented in Fig.S3(A). We modeled the hydrogel material using the permeability value (K) of 5wt.% gelMA calculated in section 2. Fig. S3(B) shows a representative velocity field around a spheroid model for ΔP = 30 mbar. The computed average flow velocities for ΔP = 20 and 30 mbar are 0.2 µm/s and 0.45 µm/s, respectively. Fig. S3(C) shows the statistics of local shear stress at the spheroid interface for the two imposed pressure gradients. The maximum fluid-induced shear stress was measured at 0.3 mPa at the spheroid interface for ΔP = 30 mbar condition. Fig. S3(D) and S3(E) show the normal stress measured at ~1750 Pa at the top spheroid interface for ΔP = 30 mbar condition and ~1250 Pa for ΔP = 20 mbar condition, respectively.

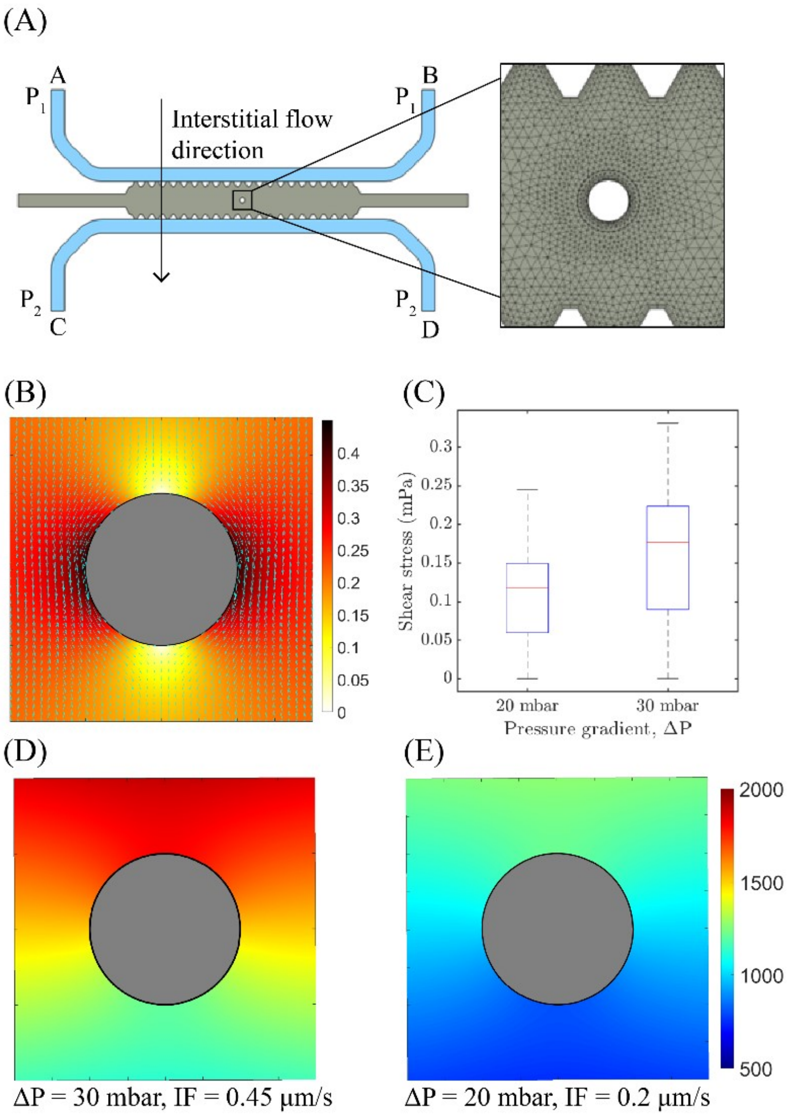

Figure S3: COMSOL simulation to compute interstitial fluid velocity and flow induced shear and normal stresses (A) 2D microfluidic model used for performing numerical simulation and the typical unstructured distribution near the spheroid model. (B) An example demonstrating the velocity field (absolute velocity) around the spheroid model at ΔP = 30 mbar (direction of flow: top to bottom, scale: μm/s). (C) IF-induced shear stress distribution at the spheroid interface under two pressure gradient conditions. (D and E) Normal stress experienced by the spheroid model at ΔP = 30 mbar and 20 mbar respectively (scale: Pa).

5. 2D microfluidic flow experiments with dual-reporter A549 cells for qPCR analyses

To further investigate the effect of flow conditions on the upregulation of Smad-signalling pathway and mesenchymal marker vimentin, we performed experiments with dual-reporter A549 cells in a 2D-microfluidic device (without matrix) subjected to flow with and without the presence of exogenous TGF-β. The 2D-flow experiments are performed in a microfluidic chip with a microchannel of dimensions 2 cm x 1 mm x 0.1 mm (length x width x height) as shown in Fig. S4, fabricated using standard soft-lithography technique, described in ESI section 2. The microchannels were coated with 20 μg/mL fibronectin (Sigma Aldrich, St. Louis, MO, USA) and kept in a humidified incubator for 6 hours before seeding dual-reporter

A59 cells at a density of 10^5 cells/mL. The microfluidic chip with seeded cells were then kept in a humidified incubator (95%) for overnight attachment to achieve a confluent 2D-monolayer. After successful cell attachment, we imaged the cells for their reporter expression (CAGA-GFP and VIM-RFP) at t = 0 hour with a Zeiss LSM 980 Confocal Microscope at 20x/NA 0.8 M27 air objective at 1024 x 1024 pixel^2 density. For GFP and RFP fluorescence, lasers with excitation wavelength of 488 nm and 543 nm were used, respectively. For the experiment, we prepared two reservoirs, one containing cell culture media supplemented with exogenous TGF-β and the other without exogenous TGF-β. To clearly identify the effect of 2D-flow, two different microfluidic devices were used, one connected to reservoir with TGF-β and the other without TGF-β. The reservoirs and microfluidic chips were placed in a humidified incubator. Next, we connected two syringes on a syringe pump (Harvard Apparatus, Elite 2000) placed outside the incubator. The tubings from the syringes were connected to the outlets of the microfluidic devices inside the incubator. The flow rate was set at 0.1 µL/minutes using the withdrawal function. We calculated the fluid-induced wall shear stress from equation (eq.2);

$$\tau = \frac{6 \times \eta \times Q}{W \times h^2} \qquad \text{(eq.2)}$$

Where η is the viscosity (in Pa.s), Q is the volumetric flow rate (m^3/s), W is the width (m) of the channel and h is the height (m) of the microfluidic channel. This flow rate creates a fluid-induced wall shear stress at 1×10^{-3} Pa. The experiment was performed for 72 hours and imaged using the same image settings described above. Simultaneously, we prepared samples under 2D-static (no-flow) conditions where A549 cells were stimulated with and without exogenous TGF-β, imaged at t = 0 and t = 72 hours. Fig. S5 (A and B) shows the merged GFP + BF and RFP + BF microscope image of reporter expression CAGA-GFP and VIM-RFP respectively of 2D cultured A549 cells at t = 72 hours for the following conditions: i) No-flow No TGF-β, ii) No-flow + TGF-β, iii) Flow – No TGF-β, iv) Flow + TGF-β. We analyzed the upregulation in fluorescence intensity for all cases and plotted the intensity measured at t = 72 hours normalized with respect to the control sample at t = 72 hours, see Fig. S5(C and D). The reporter expression in 2D-flow (without matrix) condition revealed the activity of Smad-induced CAGA-12-GFP reporter response as well as VIM-RFP indicating the abundance of vimentin protein in A549 cells. The experiment was performed in duplicates and each experiment was imaged at 3 different locations of the microfluidic channel (total n = 6 locations for each condition in 2 independent experiments). Statistical analysis on each set was performed using Student's t-test (paired two samples).

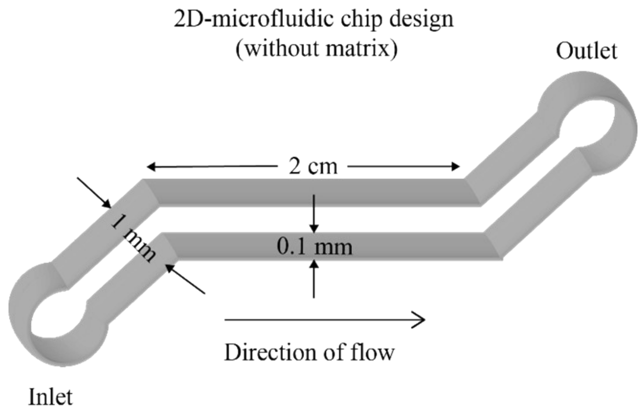

Figure S4: 2D-microfluidic chip design (with dimensions, not to scale) for experiments performed with dual

reporter A549 single cells under 2D-flow and exogenous TGF-β conditions without hydrogel matrix.

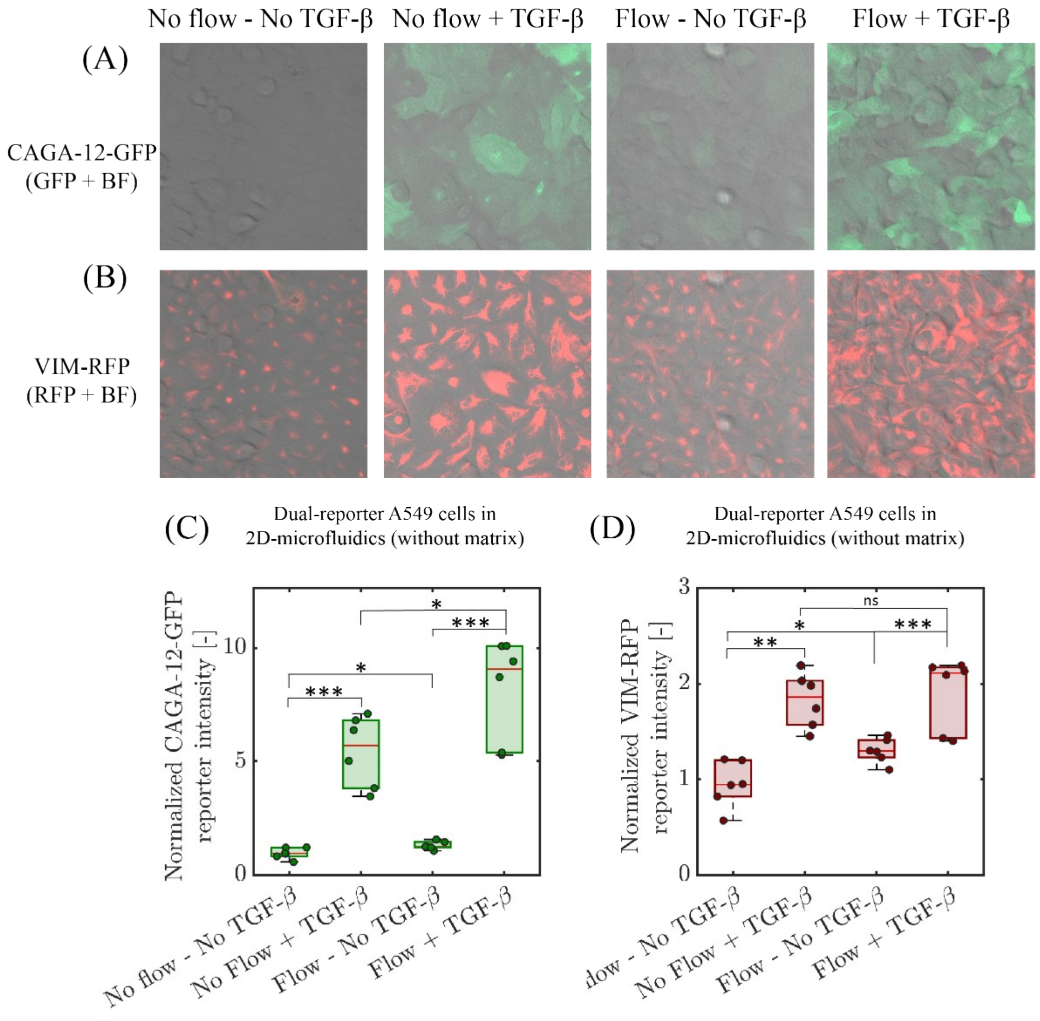

Figure S5: 2D-microfluidic (without matrix) experiments performed with dual-reporter (CAGA-12-GFP and VIM-RFP) A549 single cells. (A) Bright-field and GFP channels (merged) imaged at t = 72 hours for all experimental conditions for CAGA-12-GFP reporter expression of A549 cells. (B) Bright-field and RFP channels (merged) imaged at t = 72 hours for VIM-RFP reporter expression of A549 cells. (C) Quantification of CAGA-12-GFP fluorescence signal intensity at t = 72 hours, normalized to fluorescence intensity of No flow – No TGF-β (control) condition at t = 72 hours in 2D-microfluidics. (D) Quantification of VIM-RFP fluorescence signal intensity at t = 72 hours, normalized to No flow – No TGF-β (control) condition at t = 72 hours in 2D.

6. RNA isolation, cDNA synthesis and real time-quantitative PCR (RT-qPCR)

After the experiment, the cells from the microchannel are first extracted using trypsin for 3 minutes and diluted in fresh culture medium followed by centrifugation at 200g for 5 minutes. The supernatant is pipetted out leaving behind the cell pellet. Total RNA extraction is performed using NucleoSpin RNA II kit (Macherey-Nagel, Deuren, Germany) according to the manufacturer's instructions. cDNA was generated using the Revert Aid First-Strand cDNA synthesis mix (Thermo Fisher, Bleiswijk, The Netherlands). Quantitative PCR was performed using SYBBR GoTaq qPCR master mix (Promega, Leiden, Netherlands)

using 0.5 µM of primers. RT-qPCR was performed on the CFZ connect Real-Time PCR detection system (Bio-Rad, Hercules, CA, USA). The primers used are listed in Table S2. All experiments were performed in triplicates, target gene expression was normalized to the geometric mean (geomean) of *ARP* and *HPRT* gene expression. All quantified gene expression is normalized with No flow – No TGF-β (control) condition.

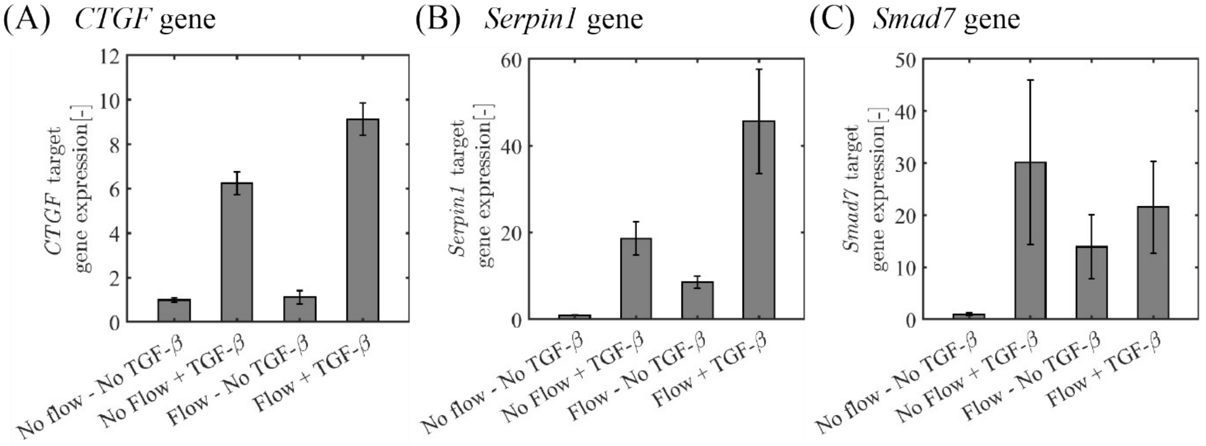

Figure S6: **TGF-β target gene analysis on 2D cultured dual-reporter A549 cells in a microfluidic chip subjected to flow (fluid-induced shear stress), exogenous TGF-β and combination of flow and exogenous TGF-β conditions, in contrast with no flow and TGF-β condition (as control condition).** qPCR results are representative of an average of three technical replicates from two independent experiments for No-Flow conditions and four independent experiments for Flow conditions performed in 2D-microfluidics (without matrix).

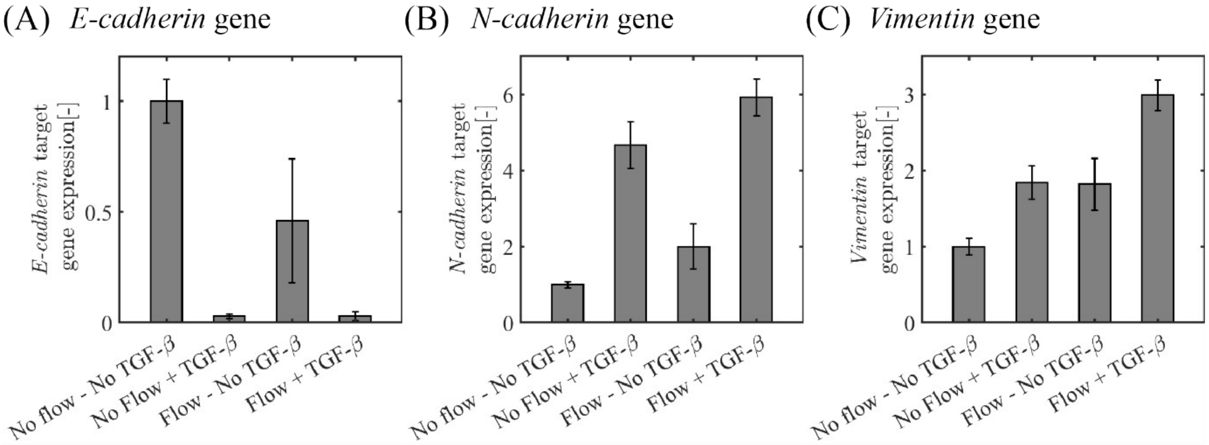

Figure S7: EMT marker gene analysis on 2D cultured dual-reporter A549 cells in a microfluidic chip subjected to flow (fluid-induced shear stress), exogenous TGF-β and combination of flow and exogenous TGF-β conditions, in contrast with no flow and TGF-β condition (as control condition). qPCR results are representative of an average of three technical replicates from two independent experiments for No-Flow conditions and four independent experiments for Flow conditions performed in 2D-microfluidics (without matrix).

**Gene**	**Forward (5' – 3')**	**Reverse (3' – 5')**
**EMT target genes**		
E-cadherin	CCCGGTATCTTCCCCGC	CAGCCGCTTTCAGATTTTCAT
N-cadherin	CAGACCGACCCAAACAGCAAC	GCAGCAACAGTAAGGACAAACATC
Vimentin	CCAAACTTTTCCTCCCTGAACC	CGTGATGCTGAGAAGTTTCGTTGA

**TGF-β target genes**		
Smad7	TCCAGATGCTGTGCCTTCC	GTCCGAATTGAGCTGTCCG
CTGF	TTGCGAAGCTGACCTGGAAGAGAA	AGCTCGGTATGTCTTCATGCTGGT
Serpin1	CACAAATCAGACGGCAGCACT	CATCGGGCGTGGTGAACTC
**Household genes**		
ARP	CACCATTGAAATCCTGAGTGATGT	TGACCAGCCGAAAGGAGAAG
HPRT	CTGGCGTCGTGATTAGTGAT	CTCGAGCAAGACGTTCAGTC

Table S2: Primer sequence for qPCR gene analysis

7. CAGA-12-GFP and VIM-RFP fluorescent reporter upregulation of A549 spheroids under No flow and IF (u_m = 0.45 µm/s, ΔP = 30 mbar) conditions at fixed exogenous TGF-β of 10 ng/mL in 3D-matrix based microfluidic chip

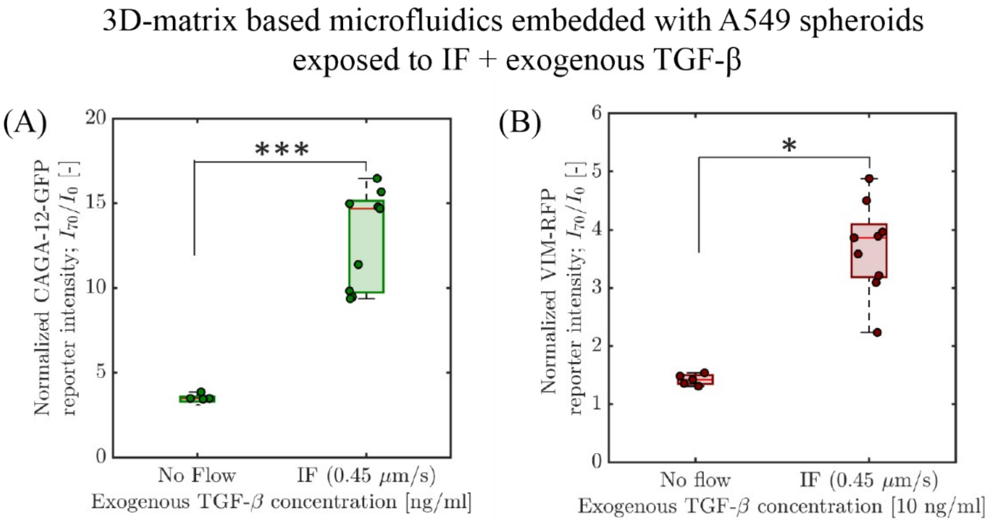

Figure S8: A) CAGA-12-GFP reporter and B) VIM-RFP reporter activity when A549 spheroids were exposed to a fixed exogenous TGF-β concentration of 10 ng/mL under no-flow and IF (u_m = 0.45 µm/s, ΔP = 30 mbar) condition in a 3D-matrix based microfluidic chip.

8. Endogenous fluorescent reporter upregulation of A549 spheroids under varying interstitial flow conditions without exogenous TGF-β in 3D-matrix based microfluidic chip

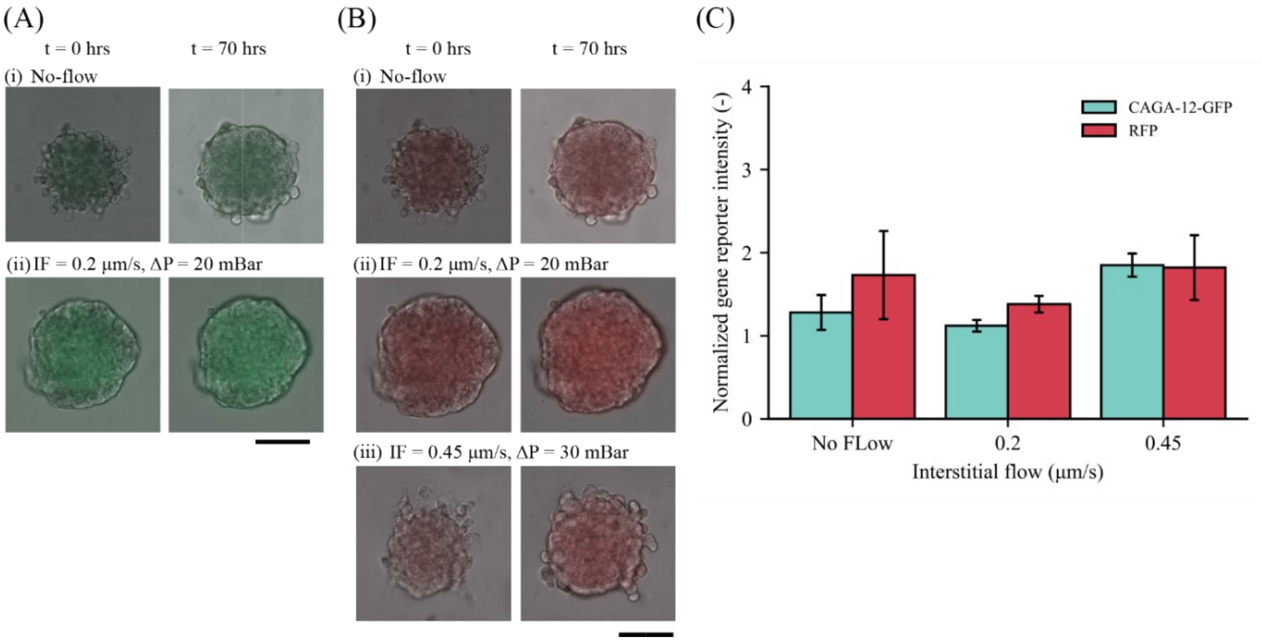

Figure S9: Endogenous CAGA-12-GFP and VIM-RFP reporter response under varying interstitial flow conditions. (A) Bright field and GFP channel superposed images of A549 spheroids showing CAGA-12-GFP reporter upregulation at t = 0 and t = 70 hours for (i) no-flow and (ii) IF (u_m = 0.2 µm/s) conditions. (B) Bright and RFP channel superposed images for vimentin upregulation in no-flow and IF conditions (scale: 100 µm). (C) Normalized CAGA-12G-GFP and VIM-RFP [I_{70}/I_0] reporter upregulation at t = 70 hours.

9. CAGA-12-GFP reporter assay using SB-4315422 (a selective small molecule TGF-β type I receptor kinase inhibitor)

i. CAGA-12-GFP reporter assay in 3D static conditions using A549 spheroids

To test for Smad-dependent CAGA-12-GFP transcriptional reporter activity in the presence of exogenous TGF-β, we performed additional validation studies for the reporter activity using TGF-β type I receptor inhibitor (SB-4315422). Experiments were performed in no-flow (static) conditions using 3D-A549 spheroids embedded in 5wt.% gelMA. A549 spheroids were first treated with SB-431542 inhibitor (10 µM) for 6 hours before adding exogenous TGF-β. The reporter activity was measured in three different conditions: i) Vehicle control (4 mm HCl in DMSO), ii) SB-431542 (10 µM) treated + exogenous TGF-β (10 ng/mL), and iii) only exogenous TGF-β (10 ng/mL), as shown in Fig. S10. Imaging was performed using Zeiss LSM 980 Confocal Microscope at 10x/NA 0.3 M27 air objective with Z-stack for GFP reporter activity at t = 0 hours (before TGF-β treatment) and t = 72 hours (after TGF-β treatment). For GFP fluorescence, laser with the excitation wavelength of 488 nm was used at 1024 x 1024 pixel^2 density. For image analysis, Z-stack images were converted to 2D-image using the Z-project function at maximum intensity in ImageJ. The fluorescence intensity at t = 72 hours of spheroids were normalized with respect to fluorescence intensity at t = 0 hour. Fig.S10 shows the relative change in expression for fluorescence intensity at t = 72 hours for all cases (n = 4 spheroids). CAGA-12-GFP transcriptional reporter activity was only observed

when A549 spheroids were stimulated only with exogenous TGF-β. On the other hand, A549 spheroids under control does not show any fluorescence upregulation due to inactivity of Smad signaling activity in the absence of exogenous TGF-β molecules. When A549 spheroids were treated with SB-431542 inhibitor, the addition of exogenous TGF-β resulted in no reporter activity. This is due to the inhibition of type I receptor kinase activity preventing Smad-dependent transcriptional reporter response.

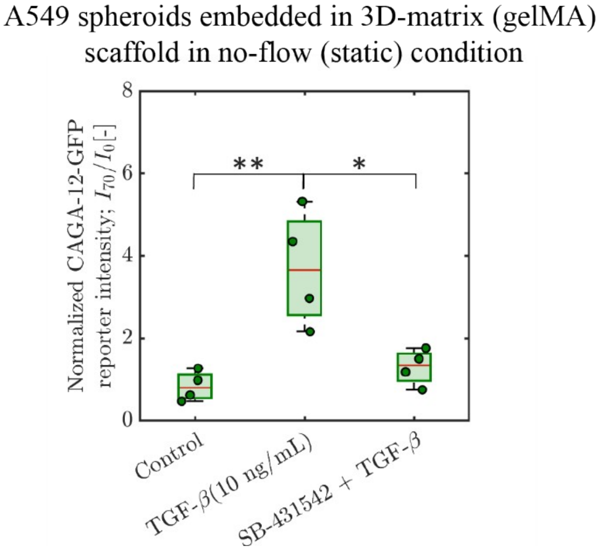

Figure S10: CAGA-12-GFP transcriptional reporter activity of A549 spheroids embedded in 5wt.% gelMA (in 3D) at t = 72 hours (n = 4 spheroids for each condition). Reporter expression is highest when stimulated with exogenous TGF-β (10 ng/mL) compared to spheroids first treated SB-431542 (TGF-β receptor kinase inhibitor) followed by exogenous TGF-β (10 ng/mL) stimulation.

ii. CAGA-12-GFP and VIM-RFP reporter assay in 2D-microfluidics under flow (without exogenous TGF- β) and SB-431542 inhibitor

To further extend these studies to explore the binding affinity of exogenous TGF- β molecules with TGF-β receptor sites, we performed additional validation studies in 2D-microfluidic device using A549 dual-reporter cells with and without TGF- β inhibitor (SB-431542). 2D-microfluidic devices were prepared by culturing dual-reporter A549 cells 2D in a microfluidic channel according to the method described in section 5 (ESI). In the first experiment, we observed CAGA-12-GFP and VIM-RFP reporter activity of A549 cells cultured in 2D-microfluidics subjected to Flow with and without SB-431542 inhibitor. For inhibitor treatment experiments, after cell-surface attachment, A549 cells were incubated in the presence of TGF-β receptor kinase inhibitor (SB-431542, 10 μM) for 6 hours. Imaging was then performed at t = 0 hr and t = 72 hours at 4 different locations of the microfluidic channel with imaging conditions described in section 5 (ESI). We plotted the CAGA-12-GFP and VIM-RFP at t = 72 hours normalized with respect to t = 0 hr. Fig. S10 (A) shows that all CAGA-12-GFP reporter activity have been blocked in A549 cells treated with SB-431542 (TGF-β receptor kinase inhibitor). Whereas, A549 cells under flow produce significant reporter expression. We observed a similar effect in VIM-RFP reporter, where cells treated with SB-431542 inhibitor showed reduced RFP reporter expression compared to cells subjected to flow, shown in Fig S10 (B). This experiment thus highlights the additional effect of flow in 2D towards Smad-dependent transcriptional reporter response.

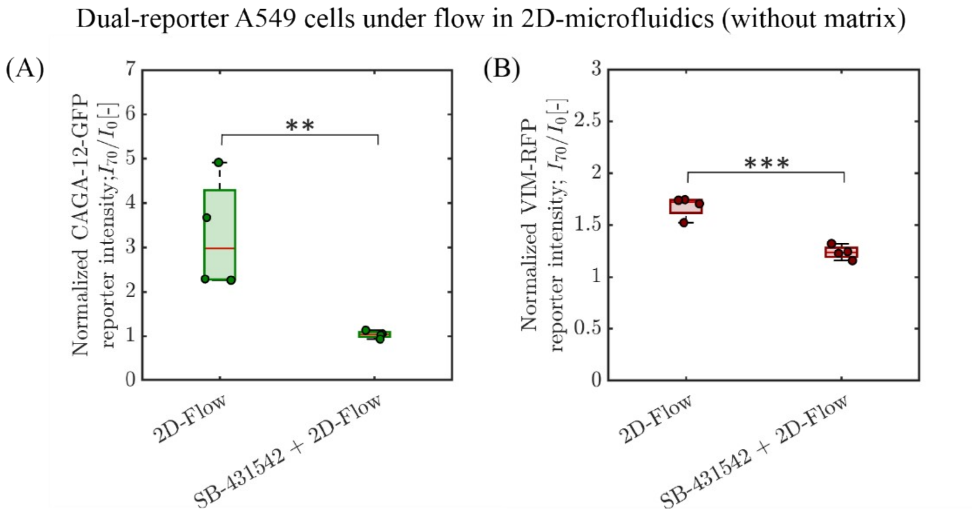

Figure S11: CAGA-12-GFP and VIM-RFP reporter expression in 2D-microfluidics (without matrix) under flow treated with and without SB-431542 (TGF-β inhibitor). A) CAGA-12-GFP reporter and B) VIM-RFP expression at t = 72 hours shows significant reporter upregulation when compared to reporter expression for A549 cells treated with SB-431542 inhibitor.

iii. CAGA-12-GFP and VIM-RFP reporter assay cultured in 2D-microfluidics under flow conditions with exogenous TGF- β and SB-431542 inhibitor

In the second experiment, A549 cells in 2D-microfluidics, CAGA-12-GFP and VIM-RFP reporter activity was quantified under flow and exogenous TGF-β with and without SB-431542 inhibitor treatment. These experiments further confirm the CAGA-12-GFP reporter activity is a consequence of the exogenous TGF-β molecules binding with TGF-β receptor sites to undergo Smad signaling. In Fig. S11 (A), A549 cells treated with SB-431542 inhibitor blocked all TGF-β receptor kinase activity in the presence of exogenous TGF-β molecules to prevent Smad-signaling response. This led to inactivity of CAGA-12-GFP transcriptional reporter upregulation. On the other hand, significant upregulation of CAGA-12-GFP activity was observed when no inhibitor treatment was performed. Similar response was obtained for VIM-RFP reporter activity, where flow and exogenous TGF-β exposure leads to higher vimentin activity, shown in Fig. S11 (B).

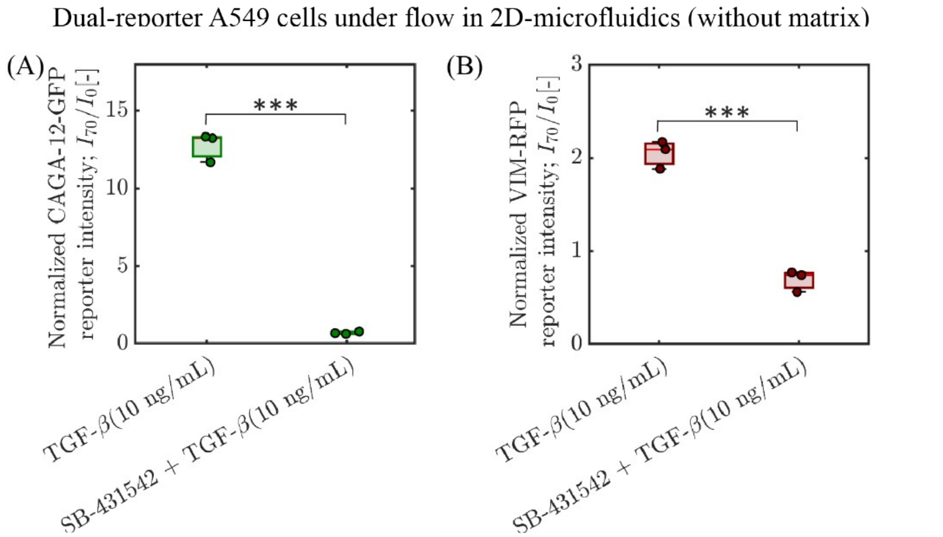

Figure S12: CAGA-12-GFP and VIM-RFP reporter expression in 2D-microfluidics with flow and exogenous TGF-β conditions treated with and without SB-431542 (TGF-β inhibitor). A) CAGA-12-GFP reporter and B) VIM-RFP expression at t = 72 hours shows significant upregulation in reporter expression when compared to reporter expression for A549 cells treated with SB-inhibitor performed in 2D-microfluidics (without matrix).

10. Evolution of exogenous TGF-β concentration and surface adsorption under varying interstitial flow condition

Based on the numerically computed velocity and pressure fields (see Section 4), we measured the temporal evolution of TGF-β concentration within the porous hydrogel matrix by solving the mass transport equations (using the Transport of Diluted Species module in COMSOL Multiphysics). To mimic the experimental conditions, the channel AB was initially filled with TGF-β (with molecular diffusivity 21.3 $\mu m^2/s$) at concentration C_0, while the remaining domains were kept at zero initial concentration. For the conditions with interstitial flow, the two inlets A and B were supplied with a continuous injection of TGF-β at concentration C_0. For the no-IF condition, the continuous injection condition was removed, and transport was fully dominated by the diffusion of TGF-β from the channel AB to the hydrogel (middle channel) with a spheroid model. The evolution of TGF-β concentration near the spheroid (at a radial distance of 0.05 mm from the interface) for various IF and no-IF conditions are shown in Fig. S14.A. Additionally, to test the penetration of exogenous TGF-β under varying IF conditions, we used FITC-labelled Dextran (20 kDa) tracer particles similar to the size of exogenous TGF-β molecules (25 kDa). In this experiment, we used wild-type A549 spheroids (unlabeled) to avoid fluorescence signal crosstalk since the excitation wavelength of CAGA-12-GFP and FITC is the same at 488 nm. A549 spheroids were embedded in 5wt.% gelMA in the microfluidic chip and operated at varying IF and no-IF conditions. Spheroids were imaged at an interval of 6 minutes for 360 minutes at 20x/NA 0.16 objective using Colibri Inverted microscope. The fluorescence intensity of the spheroids was analyzed using ImageJ. Fig.S14.B shows the fluorescence profile of A549 spheroids at varying IF and no-IF conditions. Fluorescence intensity was increased by IF as more FITC-Dextran tracer particles adsorbed or penetrated through the A549 spheroid. This experiment highlights that IF can improve the penetration of exogenous TGF-β molecules in the spheroid.

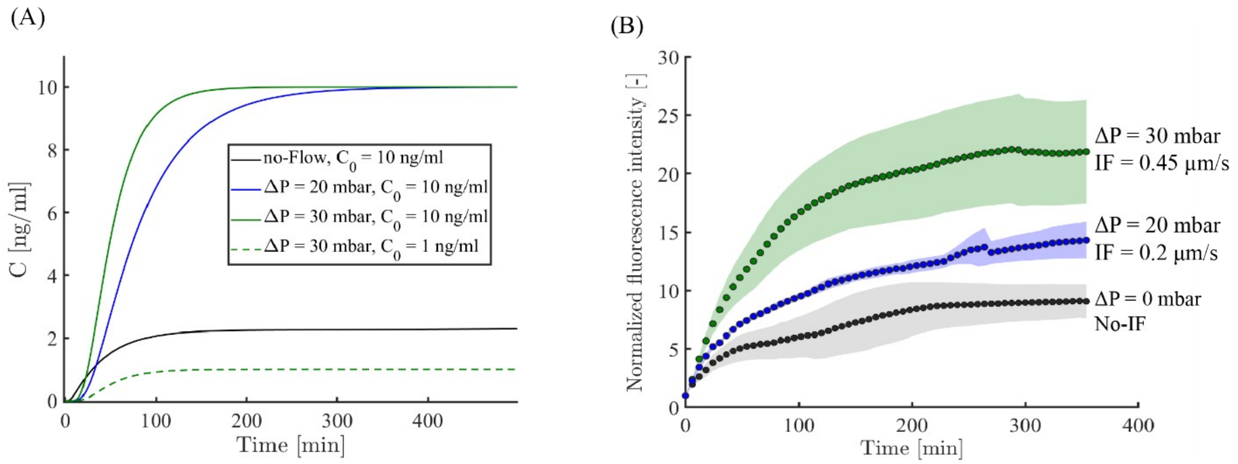

Figure S13: A) Evolution of average concentration of exogenous TGF-β (C_0 = 1 and 10 ng/mL) near a spheroid (within a radial distance of 0.05 mm) at interstitial flow conditions (ΔP = 0 (no-flow), 20 and 30 mbar). B) Normalized fluorescence intensity quantification of A549 spheroids exposed to FITC-Dextran (20kDa) tracer particles over time at different IF conditions of ΔP = 0, 20 and 30 mbar.

11. CAGA-12-GFP fluorescence profile under no-flow conditions with varying exogenous TGF-β concentrations in 3D-matrix based microfluidics.

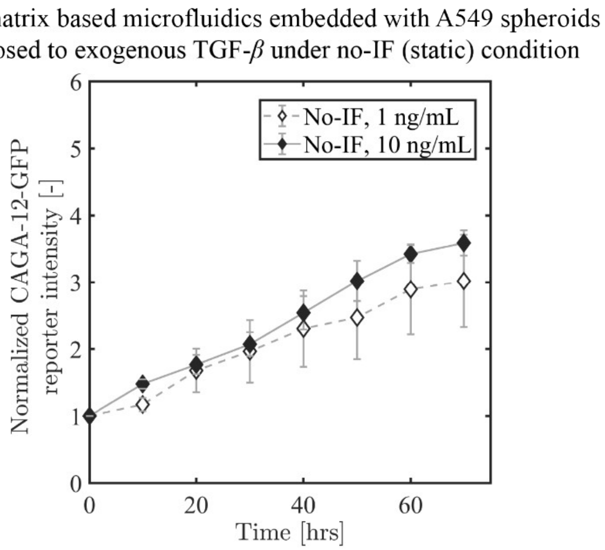

Figure S14: CAGA-12-GFP fluorescence upregulation profile of A549 spheroids embedded in gelMA under no-IF (static) condition supplemented with varying exogenous TGF-β concentration of 1 and 10 ng/mL in 3D-matrix based microfluidic chip.

12. Visualizing local heterogeneity in fluorescence expression of A549 spheroid stimulated by exogenous TGF-β

To visualize the evolution of fluorescence profile of a spheroid over the 70 hours time frame, we performed image analysis using the Polar Transformer function (ImageJ plug-in), see Fig. S15 (A). Since a spheroid in general closely represents a circular object (in 2D plane, with xy coordinate), the spheroid can

be “unwrapped” by converting this 2D-spheroid image into radius/angle coordinates (polar transformation). We do this for all spheroid images obtained at time points t = 0, 24, 48 and 70 hours, see Fig. S15(B). Using ImageJ, we measured the averaged fluorescence intensity at every angle (θ = 0 to 360 degrees) of the converted polar transformed image, see Fig. S15(B). Using a custom-made MATLAB script, we plot the average fluorescence intensity (for every θ), see Fig. S15(C).

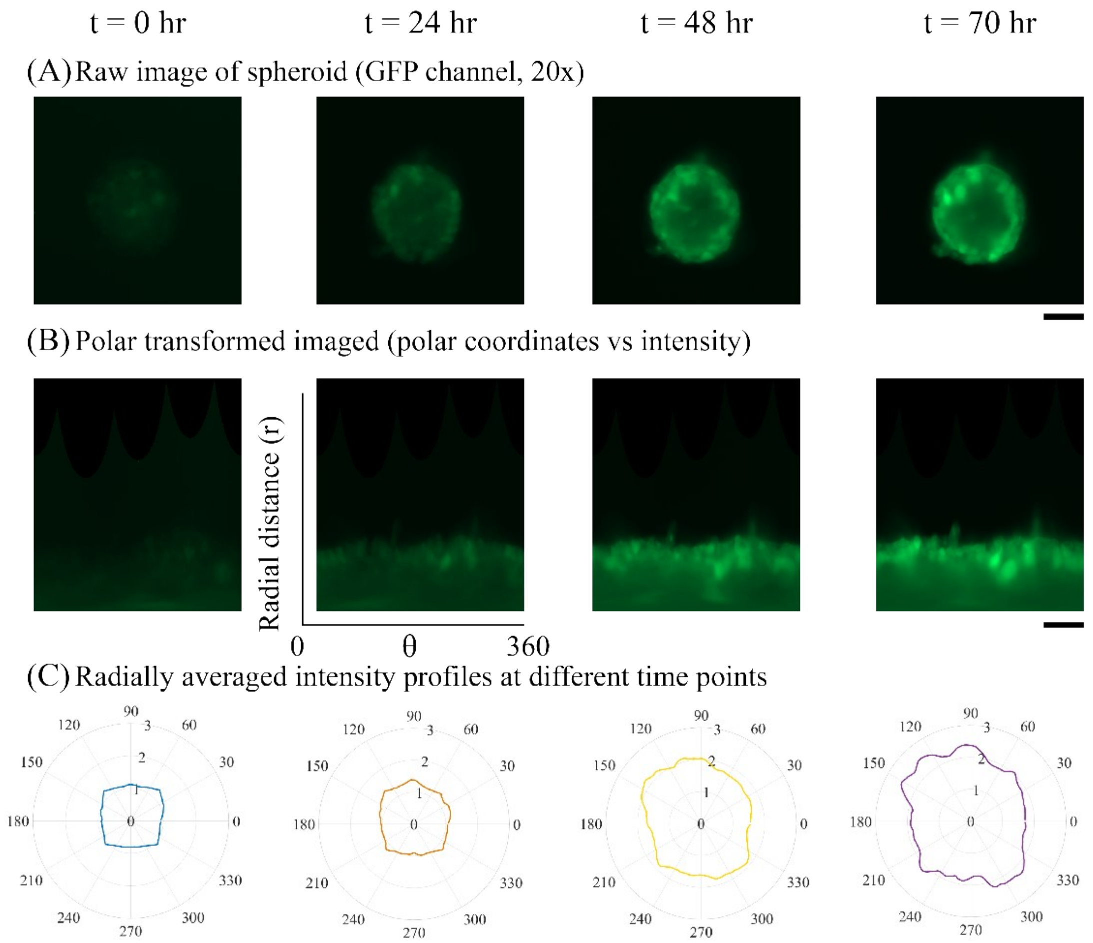

Figure S15: Methodology for polar plot analysis: (A) Raw GFP fluorescence channel image (20x) of a spheroid at different time intervals exposed to exogenous TGF-β (10 ng/mL) under no-flow conditions, scale: 100 µm. (B) Cartesian-polar transformed image obtained via Polar transformer plug-in in ImageJ, scale: 100 µm. The spheroid (circular object) is converted into an image represented in polar coordinates (θ = 0 to 360 degrees). (C) Polar plot of radially averaged fluorescence intensity at t = 0, 24, 48 and 70 hours. Each profile is normalized by the intensity at t = 0 hour to show the evolution of signal intensity in radial coordinates.

Supplementary movies

Supplementary movie S1: Time-lapse video of bright-field images of A549 spheroid embedded in gelMA in 3D-microfluidic chip exposed to IF (u_m = 0.45 µm/s, ΔP = 30 mbar) and exogenous TGF-β (10 ng/mL). Spheroids show cellular motion activity at spheroid edges over the timeframe of experiment. Most activity is visible in the top/side part of the spheroid periphery (in the direction of IF).

Supplementary movie S2: Time-lapse video of bright-field images of A549 spheroid embedded in gelMA in 3D-microfluidic chip exposed to no-IF (ΔP = 0 mbar) and exogenous TGF-β (10 ng/mL). Spheroids show minimal cellular motion at the spheroid edge over the timeframe of experiment.

References

1. Shin, Y. *et al.* Microfluidic assay for simultaneous culture of multiple cell types on surfaces or within hydrogels. *Nat. Protoc.* **7**, 1247–1259 (2012).

2. Huang, C. P. *et al.* Engineering microscale cellular niches for three-dimensional multicellular co-cultures. *Lab Chip* **9**, 1740–1748 (2009).

3. Miri, A. K., Hosseinabadi, H. G., Cecen, B., Hassan, S. & Zhang, Y. S. Permeability mapping of gelatin methacryloyl hydrogels. *Acta Biomater.* **77**, 38–47 (2018).
